# Recurrent mutation in the ancestry of a rare variant

**DOI:** 10.1093/genetics/iyad049

**Published:** 2023-03-27

**Authors:** John Wakeley, Wai-Tong (Louis) Fan, Evan Koch, Shamil Sunyaev

**Affiliations:** Department of Organismic and Evolutionary Biology, Harvard University, Cambridge, MA 02138, USA; Department of Mathematics, Indiana University, Bloomington, IN 47405, USA; Center of Mathematical Sciences and Applications, Harvard University, Cambridge, MA 02138, USA; Department of Biomedical Informatics, Harvard Medical School, Boston, MA 02115, USA; Division of Genetics, Brigham and Women’s Hospital, Harvard Medical School, Boston, MA 02115, USA; Department of Biomedical Informatics, Harvard Medical School, Boston, MA 02115, USA; Division of Genetics, Brigham and Women’s Hospital, Harvard Medical School, Boston, MA 02115, USA

**Keywords:** recurrent mutation, Ewens sampling formula, coalescent theory, human SNPs

## Abstract

Recurrent mutation produces multiple copies of the same allele which may be co-segregating in a population. Yet, most analyses of allele-frequency or site-frequency spectra assume that all observed copies of an allele trace back to a single mutation. We develop a sampling theory for the number of latent mutations in the ancestry of a rare variant, specifically a variant observed in relatively small count in a large sample. Our results follow from the statistical independence of low-count mutations, which we show to hold for the standard neutral coalescent or diffusion model of population genetics as well as for more general coalescent trees. For populations of constant size, these counts are distributed like the number of alleles in the Ewens sampling formula. We develop a Poisson sampling model for populations of varying size and illustrate it using new results for site-frequency spectra in an exponentially growing population. We apply our model to a large data set of human SNPs and use it to explain dramatic differences in site-frequency spectra across the range of mutation rates in the human genome.

Recurrent mutation has long been recognized as an important factor of evolution (Fisher 1928; Haldane 1933; Wright 1938). This is emphasized by recent analyses of single-nucleotide polymorphism (SNP) frequencies and variation of mutation rates across the human genome (Aggarwala and Voight 2016; Harpak *et al.* 2016; Seplyarskiy *et al.* 2021) describing how patterns of variation depend on the mutation rate, particularly for rare variants. By a rare variant we mean an allele, such as an alternate base at a SNP, which is observed a relatively small number of times in a large sample. Unless the mutation rate is very small, indistinguishable copies of the same allele may descend from multiple mutations. Here, we present a sampling theory for the numbers and associated frequencies of these unobserved or latent mutations in the ancestry of a rare variant.

Humans are on the low end of polymorphism levels among species (Leffler *et al.* 2012). On average, multiple mutations should be rare. In the 1000 Genomes Project data, about 1 in 1300 sites differ when two (haploid) genomes are compared, and SNPs with more than two bases segregating comprise only about 0.3% of the total SNPs observed (The 1000 Genomes Project Consortium 2015). But polymorphism rates vary by two or three orders of magnitude depending on local sequence context (Aggarwala and Voight 2016; Harpak *et al.* 2016; Seplyarskiy *et al.* 2021). Recurrent mutation is an important phenomenon for fast-mutating sites. Evidence for this can be found in the haplotype structure surrounding rare mutations (Johnson *et al.* 2022) and in the distribution of their frequencies among sites in large samples (Harpak *et al.* 2016; Seplyarskiy *et al.* 2021).

Here we focus on the latter, in particular on the site-frequency spectrum (Tajima 1989; Braverman *et al.* 1995; Fu 1995). Deviations in site-frequency spectra compared to standard predictions may be due to selection (Bustamante *et al.* 2001; Achaz 2009; Ferretti *et al.* 2017), changes in population size over time (Eldon *et al.* 2015; Liu and Fu 2015; Gao and Keinan 2016) or population structure (Gutenkunst *et al.* 2009; Städler *et al.* 2009; Kern and Hey 2017). But they may also be due to multiple mutations, i.e. to violations of the infinite-sites model assumption that each polymorphism is due to a unique mutation (Fisher 1930a; Kimura 1969, 1971; Ewens 1974; Watterson 1975).

The standard site-frequency prediction, which holds for a well-mixed population of constant large size *N* and neutral mutation rate *u* at a locus, is that the number of SNPs where a variant is found in *i* copies in a sample of size *n* should be proportional to θ/i, where θ=4Nu (Tajima 1989; Fu 1995). This dramatically underpredicts the abundance of rare variants in data from humans, which is largely due to our recent explosive population growth (Keinan and Clark 2012; Gazave *et al.* 2014; Gao and Keinan 2016), but the standard neutral model is a useful starting point for modeling recurrent mutation.

Jenkins and Song (2011) studied the occurrence of one or two mutations at a single site under the standard neutral coalescent model (Kingman 1982; Hudson 1983; Tajima 1983). They showed that if two mutations occur and are non-nested (meaning that all descendants of both mutations can be observed) there will be a shift away from rare variants and toward common ones. An earlier work focusing on the nested case is Hobolth and Wiuf (2009). Bhaskar *et al.* (2012) used a similar approach as Jenkins and Song (2011) to obtain results for one, two or three mutations, up to leading order in the mutation parameter θ. Sargsyan (2006, 2015) considered two mutations occurring at two different sites, and Jenkins *et al.* (2014) assumed that two mutations are distinguishable and yield a tri-allelic polymorphism. These latter works (Sargsyan 2006, 2015; Jenkins *et al.* 2014) allowed for variable population size following the general coalescent approach of Griffiths and Tavaré (1998). None of these works considered rare variants in particular but their predictions, especially those for non-nested mutations (Jenkins and Song 2011; Bhaskar *et al.* 2012) are helpful for understanding recurrent mutation.

Two recent large studies of human SNPs observed this predicted shift away from rare variants and toward common ones at fast-mutating sites. Harpak *et al.* (2016) surveyed about 8 million SNPs in a sample of nearly 61 000 people in version 0.2 of the Exome Aggregation Consortium database (Lek *et al.* 2016) for which data were available from other primate species. Among these, about 93.3% of these were bi-allelic, 6.5% were tri-allelic and 0.2% were quad-allelic. Harpak *et al.* (2016) took the presence of identical segregating variants in different species, ranging from chimpanzees to baboons, as indicative of a higher mutation rate at a site. Consistent with the hypothesis of multiple latent mutations at fast-mutating sites, they found fewer rare variants at bi-allelic SNPs for which the minor allele was segregating in another species, and that this effect is stronger when the other species is closer to humans.

The work we present here builds upon the second of these studies. Seplyarskiy *et al.* (2021) looked at rare variants in two datasets, one containing about 292 million variants among nearly 43 thousand individuals in TOPMed freeze 5 (Taliun *et al.* 2021) and the other containing about 182 million variants among 15 thousand individuals in gnomAD version r2.0.2 (Karczewski *et al.* 2020). Variants were divided into 192 types: each of the 3 possible base substitutions at the middle site of all 64 possible trinucleotides. A classic example of a fast-mutating site in this context would be ACG, which readily changes to ATG via a C to T transition at the CpG dinucleotide (Bird 1980; Goldman 1993). The main goals in Seplyarskiy *et al.* (2021) were to quantify how the rates of each kind of mutation vary across the genome and to partition this variation into distinct components correlated with different mutational processes.

Another aim, taken up in the Supplementary Materials of Seplyarskiy *et al.* (2021), was to correct for multiple mutations contributing to rare variants. Recurrent mutation was modeled as a multi-type Poisson process where mutations with lower sample counts occur independently at a locus to generate the appearance of higher count mutations (Desai and Plotkin 2008). The expected counts in the absence of recurrence were taken from the site-frequency spectrum at slow-mutating sites. The loss of rare variants due to recurrent mutation at fast-mutating sites was quantified for sites with up to 70 copies of a rare variant. These were considered to have descended from up to 5 mutations. Slow-mutating sites, even with rates up to the genome average in humans, should conform fairly well to the infinite-sites assumption. Resampling from these as in Seplyarskiy *et al.* (2021) is a way of controlling for the myriad unknown factors affecting the site-frequency spectrum, including growth.

In this work, we present a sampling theory for latent mutations of rare variants at each given site-frequency count in a large sample. We describe a mathematical population genetic framework for the Poisson-resampling method in Seplyarskiy *et al.* (2021) and provide closed-form analytical expressions for several quantities of interest. In short, the distributions of latent mutations and counts of rare variants depend on the expected total length of the gene genealogy of the sample, the expected lengths of branches with few descendants in the sample, and of course the mutation rate. We obtain new large-sample results for exponential growth and use these to illustrate the theory. We apply our results to a different subset of the gnomAD data than Seplyarskiy *et al.* (2021), synonymous variants observed in non-Finnish European individuals in v2.1.1, containing about 834 thousand variants at about 12.3 million sites among 57 K individuals, presorted into 97 bins based on estimates of mutation rate by the method of Seplyarskiy *et al.* (2022).

We develop and present these results in the next three sections. In “Theory for constant-size large populations,” we begin with the standard neutral coalescent or diffusion model of population genetics (Ewens 2004) and demonstrate a close connection between the Ewens sampling formula (Ewens 1972) and distributions of latent mutations. In “Theory for nonconstant populations,” we extend the results to populations which have changed in size, using the Poisson-sampling models of Watterson (1974b) and Arratia *et al.* (1992). In “Theoretical example and data application,” we compare predictions for constant size to those for exponential growth and show how the new theory can be applied to understand the effects of recurrent mutation on counts of rare variants across the range of human per-site mutation rates.

## Theory for constant-size large populations

In this section, we begin with a description of recurrent mutation via the well known predictions for allele frequencies in a population and in a sample at stationarity. We then use conditional ancestral processes to demonstrate independence of latent mutations of rare variants in a large sample and show that their numbers are distributed like the numbers of alleles in the Ewens sampling formula.

### Stationary distributions and sampling probabilities

Consider a single locus with parent-independent mutation among *K* possible alleles in a population which obeys the Wright–Fisher diffusion (Fisher 1930b; Wright 1931; Ewens 2004). Thus, the population is very large, well mixed, constant in size over time, and there is no selection. One unit of time in the diffusion process corresponds to $2N_{e}$ generations ($N_{e}$ generations for haploid species), where $N_{e}$ is the effective population size. Each gene copy or genetic lineage experiences mutations at rate θ/2 and each mutation produces an allele of type i∈{1,…,K} with probability $\pi_{i}$, with $\sum_{i}\pi_{i}=1$, independent of the allelic state of the parent. At stationarity, the joint distribution of the relative frequencies $x_{1},\ldots ,x_{K-1}$ of alleles is given by


(1) $$\phi (x_{1},\ldots ,x_{K-1})=\Gamma (\theta )\prod_{i=1}^{K}\frac{x_{i}^{\theta \pi_{i}-1}}{\Gamma (\theta \pi_{i})},$$

in which Γ(⋅) is the Gamma function, and where necessarily $x_{K}=1-\sum_{i<K}x_{i}$ (Wright 1931, 1949).

Conditional on the population frequencies ($X_{1},\ldots ,X_{K})$, the sample counts of alleles ($N_{1},\ldots ,N_{K})$ are multinomially distributed. A sample of size *n* taken from the population contains $n_{1},\ldots ,n_{K-1}$ copies of alleles 1 through K−1, and necessarily $n_{K}=n-\sum_{i<K}n_{i}$ copies of allele *K*, with probability


(2) $$\;\begin{array}{rl} \;p(n_{1},\ldots ,n_{K-1};n) & \equiv P[N_{1}=n_{1},\ldots ,N_{K-1}=n_{K-1};n]\\ & =\left(\begin{array}{c} n\\ n_{1}\cdots n_{K} \end{array}\right)E[X_{1}^{n_{1}}\cdots X_{K-1}^{n_{K-1}}] \end{array}$$

(3) $$\;=\left(\begin{array}{c} n\\ n_{1}\cdots n_{K} \end{array}\right)(\theta^{\left(n\right)}{)}^{-1}\prod_{i=1}^{K}(\theta \pi_{i}{)}^{\left(n_{i}\right)}$$

for $n_{i}\in \{0,1,\ldots ,n\}$ constrained by $\sum_{i}n_{i}=n$ and where $k^{\left(r\right)}$ denotes the Pochhammer function or rising factorial k(k+1)⋯(k+r−1) with $k^{\left(0\right)}=1$. The shorthand defined in (2) is used extensively in what follows.

In applications to DNA, K=4 and a sample at a given site would contain counts $n_{1}$, $n_{2}$, $n_{3}$, $n_{4}$ of each of the four nucleotides. The assumption of parent-independent mutation which leads to the relatively simple expressions (1) and (3) is unrealistic for DNA, but its results are useful in the case of rare variants in very large samples. In this case, it is likely that the common variant, allele 4 say, represents the ancestral state of the entire sample and that rare variants (alleles 1, 2 and 3) are due to recent mutations from the common variant. Then the mutation parameter $\theta \pi_{i}$ for i∈{1,2,3} captures the production of type-*i* rare alleles in a specific ancestral background (allele 4).

An instructive special case is K=2, where we have


(4) $$\phi (x)=\frac{\Gamma (\theta )}{\Gamma (\theta \pi_{1})\Gamma (\theta \pi_{2})}x^{\theta \pi_{1}-1}(1-x{)}^{\theta \pi_{2}-1}$$

for the stationary distribution of the frequency of type 1 in the population Wright (1931), and


(5) $$p(n_{1};\;n)=\left(\begin{array}{c} n\\ \;n_{1} \end{array}\right)\frac{(\theta \pi_{1}{)}^{\left(n_{1}\right)}(\theta \pi_{2}{)}^{\left(n-n_{1}\right)}}{\theta^{\left(n\right)}}$$

for the sampling probability, i.e. that a sample of size *n* contains $n_{1}$ copies of allele 1 and $n_{2}=n-n_{1}$ copies of allele 2. Any two-allele mutation model can be described as a parent-independent model, but this is not so in general for K>2.

**Fig. 1.**
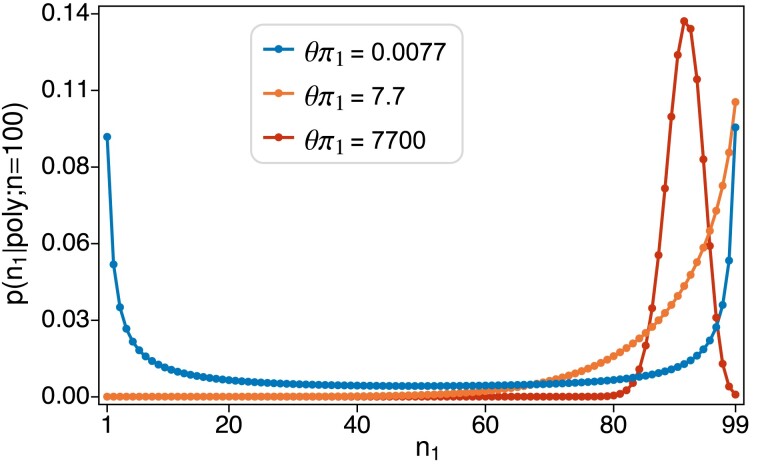
Sample frequency distribution $p(n_{1};n)$ for n=100, with $\pi_{1}=10\pi_{2}$ and three values of θ. The smallest θ was chosen so that $\theta \pi_{2}=1/1300∼0.00077$, i.e. the human average value. Probabilities are normalized to sum to one, i.e. conditioned on the sample being polymorphic ($1\leq n_{1}\leq 99$).

Figure 1 shows how the sample frequency distribution $p(n_{1};n)$ in (5) depends on the mutation rate for a pair of alleles which differ by an order of magnitude in mutation rate. Three value of θ are shown, with the small value chosen so that the mutation rate for allele 2 ($\theta \pi_{2}$) is equal to the human average of about 1/1300 (The 1000 Genomes Project Consortium 2015) and the mutation rate for allele 1 ($\theta \pi_{1}$) is ten times that. When θ is small, the distribution is U-shaped and nearly symmetric, given that the sample is polymorphic. When θ is around one, the distribution becomes J-shaped (or L-shaped if $\pi_{1}<\pi_{2}$). When θ is large, the distribution has a peak around $\pi_{1}$. Graphs of ϕ(x) (not shown) display these same shapes, and $p(n_{1};n)$ will be very close to ϕ(x)dx when *n* is large.

#### Relationship to infinite-sites frequency spectra

We use θ for the per-site mutation parameter. In a collection of *L* total sites at which (5) holds, the finite-sites version of the site-frequency spectrum (i.e. the expected number of sites with $n_{1}$ copies of allele 1 and $n_{2}$ copies of allele 2) is given by the product $Lp(n_{1};n)$. Note, these expected numbers of sites do not depend on the rate of recombination, whereas the variances among sites and covariances between sites do (Kaplan and Hudson 1985).

Infinite-sites mutation models may be obtained as limits of finite-sites models as *L* tends to infinity with the total mutation parameter Lθ remaining finite. So when θ is small, we expect finite-sites results to be close to the usual (infinite-sites) predictions from the diffusion model (Ewens 1979, 2004) or the coalescent model (Fu 1995). Finite-sites models distinguish between kinds of mutations, subject to different mutation pressures, whereas infinite-sites models implicitly treat all mutations the same.

From Ewens (1979) equation (8.18) or Ewens (2004) equation (9.18)—see also Wright (1938) equation (16)—the expected number of sites segregating in the population with frequencies between *x* and x+dx under the infinite-sites model is proportional to 1/x. For comparison to (4) we may write


(6) $$\phi_{ISM}(x)\propto \frac{\theta \pi_{1}}{x}$$

for a single site (θ small) approximately under the standard infinite-sites mutation model. For comparison with (5), we have


(7) $$p_{ISM}(n_{1};n)\propto \frac{\theta \pi_{1}}{n_{1}}$$

for the approximate single-site probability that there are $n_{1}$ type-1 alleles in a sample of size *n*. Equation (7) has the same form as the usual infinite-sites site-frequency spectrum (Fu 1995) but here it is for a specific mutant (allele 1) with a specific ancestral type (allele 2 in the two-allele model).

From (4) and (5) with θ small we have


(8) $$\phi (x)=\pi_{2}\frac{\theta \pi_{1}}{x}+\pi_{1}\frac{\theta \pi_{2}}{1-x}+O(\theta^{2})$$

and


(9) $$p(n_{1};n)=\pi_{2}\frac{\theta \pi_{1}}{n_{1}}+\pi_{1}\frac{\theta \pi_{2}}{n_{2}}+O(\theta^{2})$$

for $n_{1}\in \{1,\ldots ,n-1\}$. The diffusion result (4) does not admit atoms of probability at x=0 or x=1—see section 10.7 of Ewens (2004) for discussion—but we can interpret (8) intuitively as follows. If θ is close to zero, most of the time the population will be fixed, containing only allele 1 with probability $\pi_{1}$ and only allele 2 with probability $\pi_{2}$. Mutants of type 2 and type 1 are introduced with rates $\theta \pi_{2}$ and $\theta \pi_{1}$ in these two backgrounds, respectively. Then the leading terms in (8) represent a mixture of two infinite-sites models like (6) with the constants of proportionality specified. Equation (9) has an identical interpretation, as a mixture of two infinite-sites site-frequency spectra. These are the key principles of the boundary mutation model (Vogl and Clemente 2012; Vogl *et al.* 2020).

Although no closed-form expression like (1) is available except under parent-independent mutation, Burden and Tang (2016, 2017) have shown that the stationary densities for pairs of alleles under general mutation models take forms identical to (8) when θ is small; see equation (21) in Burden and Tang (2017). See also Schrempf and Hobolth (2017). Similarly from a coalescent analysis of general *K*-alleles mutation, Bhaskar *et al.* (2012) obtained leading order terms for sampling probabilities with forms identical to (9) when θ is small and samples contain just two alleles. For K=2, the result from Theorem 1 of Bhaskar *et al.* (2012) is identical to (9).

### Mutation and the frequencies of rare sample variants

Our goal here is to understand how the frequency spectra of rare variants depend on θ and on the number of mutation events in the ancestry of the sample under the standard neutral coalescent or diffusion model of population genetics which assumes constant population size (Ewens 2004). We first describe an ancestral process for the sample, then focus on rare variants in a large sample to obtain predictions about latent mutations.

#### A conditional ancestral process for rare variants

Here, we focus on ordered samples because the calculations are more intuitively related to the familiar rates of events in the ancestral coalescent process. The results do not depend on the order and so apply equally to ordered and unordered samples. Using the subscript “o” for ordered and writing $p_{o}(n_{1},\ldots ,n_{K})$ in place of $p_{o}(n_{1},\ldots ,n_{K-1};n)$ to facilitate the calculations, we have


(10) $$p_{o}(n_{1},\ldots ,n_{K})=(\theta^{\left(n\right)}{)}^{-1}\prod_{i=1}^{K}(\theta \pi_{i}{)}^{\left(n_{i}\right)}$$

which differs from the sampling probability in (3) only by the multinomial coefficient, or the number of ways a sample containing allele counts $n_{1},\ldots ,n_{K}$ can be ordered.

Equation (10) is suggestive, as are (3) and (5), that the sampling structure of the $n_{i}$ copies of allele *i* may be related to the Ewens sampling formula (Ewens 1972). Specifically, from the fact that


(11) $$(\theta \pi_{i}{)}^{\left(n_{i}\right)}=\sum_{k_{i}=1}^{n_{i}}\left|S_{n_{i}}^{\left(k_{i}\right)}\right|(\theta \pi_{i}{)}^{k_{i}},$$

where $\left|S_{n_{i}}^{\left(k_{i}\right)}\right|$ is an (unsigned) Stirling number of the first kind, we might guess that there is a latent variable $k_{i}$ which is the number of mutations giving rise to the $n_{i}$ copies of allele *i*. As in the usual application of the Ewens sampling formula, in contrast to the total possible number of type-*i* mutations in the ancestry of the sample, these latent mutations are just those $k_{i}\in \{1,\ldots ,n_{i}\}$ most recent ones which produced the observed alleles.

That is, based on (10) and (11), we suppose that the joint probability of the sample counts $n_{1},\ldots ,n_{K}$ and their numbers of latent mutations $k_{1},\ldots ,k_{K}$ is given by


(12) $$p_{o}(k_{1},\ldots ,k_{K},n_{1},\ldots ,n_{K})=(\theta^{\left(n\right)}{)}^{-1}\prod_{i=1}^{K}\left|S_{n_{i}}^{\left(k_{i}\right)}\right|(\theta \pi_{i}{)}^{k_{i}},$$

and therefore that the probability of $k_{1},\ldots ,k_{K}$ conditional on $n_{1},\ldots ,n_{K}$ is given by


(13) $$p(k_{1},\ldots ,k_{K}|n_{1},\ldots ,n_{K})=\prod_{i=1}^{K}\frac{\left|S_{n_{i}}^{\left(k_{i}\right)}\right|(\theta \pi_{i}{)}^{k_{i}}}{(\theta \pi_{i}{)}^{\left(n_{i}\right)}}$$

which applies to both ordered and unordered samples.

We show that (13) is true using the ancestral-process approach of Griffiths and Tavaré (1994a, 1994b). If sampling probabilities like (3) or (10) are known, this approach can be used to describe the conditional ancestral process of a sample given its allelic types (Slade 2000a, 2000b; Fearnhead 2001, 2002; Stephens and Donnelly 2003; Baake and Bialowons 2008). Following our analysis of (13) for arbitrary $\left(n_{1},\ldots ,n_{K}\right)$, we describe a large-*n* approximation in which allele *K* is the overwhelmingly common type and 1 through K−1 are the rare variants.

The conditional ancestral process has the same total rate of mutation and coalescence as the unconditional process, n(θ+n−1)/2. Lineages which must be of type *i* in the sample experience type-*i* mutations at rate $n_{i}\theta \pi_{i}/2$ and type-*i* coalescent events at rate $n_{i}(n_{i}-1)/2$, but with additional weights proportional to the probability of $\left(n_{1},\ldots ,n_{K}\right)$ given each event. All other events have rates equal to zero because the sample could not be $\left(n_{1},\ldots ,n_{K}\right)$ if they occurred. To obtain (13), we follow ancestral lineages only back to the first mutation event they experience. The probability of a type-*i* mutation event is


(14a) $$\frac{n_{i}\theta \pi_{i}p_{o}(\ldots ,n_{i}-1,\ldots )}{n(\theta +n-1)p_{o}(n_{1},\ldots ,n_{K})}=\frac{n_{i}}{n}\frac{\theta \pi_{i}}{\theta \pi_{i}+n_{i}-1},$$

and the probability of a type-*i* coalescent event is


(14b) $$\frac{n_{i}(n_{i}-1)p_{o}(\ldots ,n_{i}-1,\ldots )}{n(\theta +n-1)p_{o}(n_{1},\ldots ,n_{K})}=\frac{n_{i}}{n}\frac{n_{i}-1}{\theta \pi_{i}+n_{i}-1},$$

where we have used (10) to obtain the results on the right. Whether mutation or coalescence occurs, the number of type *i* lineages decreases by one: $n_{i}\to n_{i}-1$. This ancestral process continues until there are no un-mutated ancestral lineages, that is until $n_{i}=0$ for all i∈{1,…,K}.

To this we add a mutation counting process which starts with $k_{i}=0$ for all i∈{1,…,K} then has $k_{i}\to k_{i}+1$ whenever a mutation occurs on a type-*i* ancestral lineage. Equations (14a) and (14b) show that each event in the ancestral process includes two sub-events: a choice of the allelic type involved then a choice between mutation and coalescence. Depending on $\left(n_{1},\ldots ,n_{K}\right)$, the $n=\sum_{i}n_{i}$ choices of allelic type will result in a random ordering of events among types. But for every ordering, the series of choices between mutation and coalescence within allelic type *i* depends only on $n_{i}$ (and $\theta \pi_{i}$) and is independent of what happens in the ancestry of allele j≠i. The number of mutations of type *i* is the sum of $n_{i}$ Bernoulli random variables with success probabilities $\theta \pi_{i}/(\theta \pi_{i}+j-1)$ for *j* from $n_{i}$ down to 1. The number of latent mutations counted in this way will be distributed like the number of alleles in the Ewens sampling formula—see Arratia *et al.* (1992) and Arratia and Tavaré (1992)—with mutation parameter $\theta \pi_{i}$ for allele *i*, and these counts will be independent among alleles as in (13).

We use this conditional ancestral process below but here note its close relationship to models of lines of descent (Griffiths 1980; Watterson 1984). In particular, (13) is included in equation (3.3) and Theorem 4 of Donnelly (1986), who extended Watterson’s lines-of-descent model to the case of *K*-allele, parent-independent mutation. See also Donnelly and Tavaré (1987). Equation (3.3) in Donnelly (1986) in fact shows that if we were to keep track of the numbers of descendants of each latent mutation, the full Ewens sampling formula would give their distribution in the sample.

Before describing a large-*n* approximation for rare variants, we also note that latent mutations reckoned as in (13) include what Donnelly (1986) called ‘spurious mutations to one’s own type’ and Baake and Bialowons (2008) called ‘empty mutations’. These are a modeling artifact not only of parent-independent mutation models but of general mutation models as they are typically implemented (Jenkins and Song 2011; Bhaskar *et al.* 2012; Jenkins *et al.* 2014; Burden and Tang 2017; Burden and Griffiths 2019). Empty mutations have no empirical significance and should not be counted as mutations. To deal with them, we must keep track of the ancestral types of lineages when they experience mutations. We can do using the identity


(15) $$p_{o}(\ldots ,n_{i}-1,\ldots )=\sum_{\;j=1}^{K}p_{o}(\ldots ,n_{i}-1,\ldots ,n_{j}+1,\ldots )$$

which decomposes our previously generic type-*i* mutations according to their ancestral types j∈{1,…,K}. A mutation is empty when j=i.

In our large-*n* approximation, we take *K* to be the overwhelmingly common allelic type in the sample and 1 through K−1 to be the rare variants. Our goal is to model latent mutations in the ancestry of the rare variants, so we use (15) only for i∈{1,…,K−1}. For the common allele *K*, we instead lump (14a) and (14b) together and record both mutation and coalescence as $n_{K}\to n_{K}-1$. Making these changes to (14a) and (14b), and again using (10) to simplify ratios of sampling probabilities, the conditional ancestral process for a sample with state $\left(n_{1},\ldots ,n_{K}\right)$ jumps to state $\left(\ldots ,n_{i}-1,\ldots ,n_{j}+1,\ldots \right)$ for i,j≠K with probability


(16a) $$\frac{n_{i}}{n}\frac{\theta \pi_{i}(\theta \pi_{j}+n_{j}-\delta_{ij})}{\left(\theta +n-1)(\theta \pi_{i}+n_{i}-1\right)},$$

to state $\left(\ldots ,n_{i}-1,\ldots ,n_{K}+1\right)$ for i≠K with probability


(16b) $$\frac{n_{i}}{n}\frac{\theta \pi_{i}(\theta \pi_{K}+n_{K})}{\left(\theta +n-1)(\theta \pi_{i}+n_{i}-1\right)},$$

to state $\left(\ldots ,n_{i}-1,\ldots \right)$ for i≠K with probability


(16c) $$\frac{n_{i}}{n}\frac{n_{i}-1}{\theta \pi_{i}+n_{i}-1},$$

and to state $\left(\ldots ,n_{K}-1\right)$ with probability


(16d) $$\frac{n_{K}}{n}$$

where we have used Kronecker’s delta to accommodate empty mutations, i=j in (16a). Equation (16a) includes both empty and nonempty mutations, but only ones where the ancestral type is also rare. Nonempty mutations where the ancestral type is the common type *K* are in (16b). This classification of mutations by ancestral type does not change the probabilities of coalescence, so (16c) only differs from (14b) by the absence of type-*K* coalescent events which are now in (16d).

If $n_{K}$ is large compared to $n_{1}$ through $n_{K-1}$, then $n=\sum_{i}n_{i}\approx n_{K}$. The probabilities in (16a) will be $O(1/n_{K}^{2})$, those in (16b) and (16c) will be $O(1/n_{K})$, and the one in (16d) will be O(1). Empty mutations and other mutations with rare-variant ancestors will become negligible as $n_{K}$ grows for fixed $n_{1}$ through $n_{K-1}$. Keeping only terms of $O(1/n_{K})$ and larger gives an approximate, large-*n* ancestral process with total rate $n(\theta +n-1)/2\approx n_{K}^{2}/2$ and jumps, for i∈{1,…,K−1}, from state $\left(n_{1},\ldots ,n_{K}\right)$ to state $\left(\ldots ,n_{i}-1,\ldots ,n_{K}+1\right)$ with probability


(17a) $$\frac{n_{i}}{n_{K}}\frac{\theta \pi_{i}}{\theta \pi_{i}+n_{i}-1},$$

to state $\left(\ldots ,n_{i}-1,\ldots \right)$ with probability


(17b) $$\frac{n_{i}}{n_{K}}\frac{n_{i}-1}{\theta \pi_{i}+n_{i}-1},$$

and to state $\left(\ldots ,n_{K}-1\right)$ with probability


(17c) $$1-\frac{\sum_{i=1}^{K-1}n_{i}}{n_{K}}.$$

This process is dominated by (17c), that is by events on lineages ancestral to the common allele *K*, which decrease the number of these but leave the counts of rare-allele lineages unchanged. Although we are not tracing the details of common-allele ancestry, we note that the overwhelming majority of these events will be coalescent events, since their rate is approximately equal to the total rate $∼n_{K}^{2}/2$. The next most frequent will be empty mutation events at rate $O(n_{K})$, followed by common-allele mutation events with rare-allele ancestors at rate O(1).

When one of the rarer events occurs in the ancestral process, it involves allele *i* with probability $n_{i}/n_{K}$, then is either a mutation event from a common allele as in (17a) or a coalescent event as in (17b). This process for the rare variants i∈{1,…,K−1} has the same form as that found for all variants and all mutations in (14a) and (14b). Then by the same logic as before, the number of (now nonempty) latent mutations in the ancestry of the rare variants will be distributed like the number of alleles in the Ewens sampling formula, independently and with mutation parameter $\theta \pi_{i}$ for allele i∈{1,…,K−1}. In addition if we were to keep track of the counts of each mutation’s descendants among the $n_{i}$ copies of rare variant *i* in the sample, then because every pair of type-*i* lineages is equally likely to be the one which coalesces when a type-*i* coalescent event occurs, the distribution of these counts should be given by the full Ewen’s sampling formula (Ewens 1972; Kingman 1982; Donnelly 1986; Arratia and Tavaré 1992; Arratia *et al.* 1992, 2016).

The events involving the common allele in (17c) occur very quickly. But since only a fixed number of events involving rare alleles are required to resolve the ancestry of latent mutation and coalescence, the approximation remains accurate until all the rare-allele events have happened, if $n_{K}$ is large enough. In Appendix section “Time-dependent conditional ancestral process,” we study the joint distribution of the times of events among the rare alleles and the numbers of common-allele ancestors when these rare-allele events occur. Focusing on the case of two alleles for simplicity, if $T_{i}$ is the time back to the *i*th event involving the rare allele 1, we have


(18) $$E[T_{1}]\approx \left\{ \begin{array}{cc} \frac{2\log\;(n_{2})}{n_{2}} & \mathrm{if}\;n_{1}=1\\ \frac{2}{n_{2}(n_{1}-1)} & \mathrm{if}\;n_{1}>1 \end{array}\right.$$

which in either case tends to zero as $n_{2}$ tends to infinity. Further, if $N_{2}(T_{i})$ is the random number of type-2 ancestral lineages left at the *i*th event involving the rare allele 1, we have


(19) $$E[N_{2}(T_{i})]\approx n_{2}\frac{n_{1}-i+1}{n_{1}+1}$$

suggesting that, despite the rapid decrease of common-variant lineages, the approximation can hold until the entire ancestry of latent mutation and coalescence is resolved.

Even for the largest rare-variant site-frequency count considered in Seplyarskiy *et al.* (2021), there will still be >1200 common-variant lineages left on average at $T_{70}$ for the TOPMed data ($n_{2}∼86,000$) and >400 left for the gnomAD data ($n_{2}∼30,000$). In section “Application to human SNP data,” we consider site-frequency counts up to 40 for synonymous exonic sites in gnomAD with many fewer SNPs but a larger sample size ($n_{2}∼114,000$) and in this case there should be about 2780 common-variant lineages left at $T_{40}$ when the entire ancestry of latent mutation and coalescence among the rare variants is resolved.

In sum, rare alleles in a large sample will quickly coalesce and mutate. Their ancestors will be common alleles. If $k_{i}\in \{1,\ldots ,n_{i}\}$ is the number of these latent mutations in the ancestry of allele i∈{1,…,K−1}, then from the rates of mutation and coalescence in (17a) and (17b) we have


(20) $$p(k_{1},\ldots ,k_{K-1}|n_{1},\ldots ,n_{K-1};n\;\mathrm{large})\approx \prod_{i=1}^{K-1}\frac{\left|S_{n_{i}}^{\left(k_{i}\right)}\right|(\theta \pi_{i}{)}^{k_{i}}}{(\theta \pi_{i}{)}^{\left(n_{i}\right)}}.$$

Latent mutations of different rare variants are independent and distributed like the numbers of alleles in the Ewens sampling formula, each with its own mutation parameter.

#### Latent mutations and sample counts of rare alleles

Our goal in this section is to understand how predictions about the counts of rare variants, and hence about their site-frequency spectra, depend on the number of latent mutations and the mutation rate. In anticipation of “Application to human SNP data,” we focus on the marginal count of just one rare variant, which we arbitrarily call allele 1. From (20) we have


(21) $$p(k_{1}|n_{1};n\;\mathrm{large})\approx \frac{\left|S_{n_{1}}^{\left(k_{1}\right)}\right|(\theta \pi_{1}{)}^{k_{1}}}{(\theta \pi_{1}{)}^{\left(n_{1}\right)}},\;k_{1}\in \{1,\ldots ,n_{1}\}$$

which we note holds for any *K*. Here we let K=2 for simplicity.

To understand how the mutation rate influences the count of a rare variant, we apply the result for ratios of gamma functions with a common large parameter, 6.1.47 in Abramowitz and Stegun (1964) or equation (1) in Tricomi and Erdélyi (1951), to the terms involving *n* in (5) to obtain


(22) $$p(n_{1};n)=\frac{(\theta \pi_{1}{)}^{\left(n_{1}\right)}}{n_{1}!}e^{-\theta \pi_{1}\log\;(n)}\frac{\Gamma (\theta )}{\Gamma (\theta \pi_{2})}\left[1+O\left(\frac{1}{n}\right)\right],$$

in which we have used $n^{-\theta \pi_{1}}=e^{-\theta \pi_{1}\log\;(n)}$ to make a connection with the underlying coalescent tree or gene genealogy. Specifically, $\theta \pi_{1}\sum_{i=1}^{n-1}1/i$ is the expected number of type-1 mutations on the gene genealogy of a sample of size *n*, and for large *n* this is approximately equal to $\theta \pi_{1}(\log\;(n)+\gamma )$ where γ=0.5772… is Euler’s constant. In “Theory for nonconstant populations” we explore this connection in detail and explain the additional constants of proportionality in (22) after finding an analogous result for the general coalescent trees of Griffiths and Tavaré (1998).

Site-frequency spectra are typically defined as the proportion of segregating sites in each possible count in the sample (Braverman *et al.* 1995) or equivalently as the probability that a single mutation is in each possible count given that it is polymorphic in the sample (Griffiths and Tavaré 1998; Nielsen 2000). So, to understand how $n_{1}$ depends on $\theta \pi_{1}$, we may ignore the constants of proportionality in (22) and focus on


(23) $$p(n_{1};n\;\mathrm{large})\propto \frac{(\theta \pi_{1}{)}^{\left(n_{1}\right)}}{n_{1}!}.$$

**Fig. 2.**
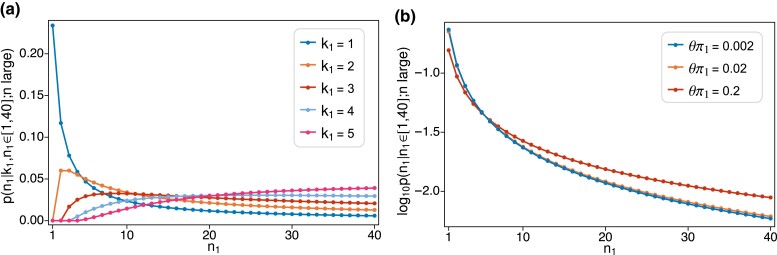
a) Shows the probability of observing $n_{1}$ copies of allele 1 in a large sample given these are produced by $k_{1}$ mutations. b) Shows the $\log_{10}$-probability of observing $n_{1}$ copies of allele 1 in a large sample for three different values of $\theta \pi_{1}$. In both panels, probabilities are normalized to sum to one, that is conditioned on the event that $n_{1}\in \{1,2,\ldots ,40\}$.

Then using (23) together with (21), we have


(24) $$p(n_{1}|k_{1};n\;\mathrm{large})\propto \frac{\left|S_{n_{1}}^{\left(k_{1}\right)}\right|}{n_{1}!}$$

for the dependence of the rare-variant count, $n_{1}$, on the number of latent mutations, $k_{1}$, relevant to the site-frequency spectrum. Figure 2 shows site-frequency spectra computed using (23) and (24), and conditioning on the event that $n_{1}\in \{1,2,\ldots ,40\}$.

Figure 2a shows the dependence on the number of latent mutations. When all copies descend from a single mutation ($k_{1}=1$), the usual predictions from the infinite-sites model hold. Thus if we put $|S_{n_{1}}^{\left(1\right)}|=(n_{1}-1)!$ in (24), then consistent with (7) we have


$$p(n_{1}|k_{1}=1;n\;\mathrm{large})\propto \frac{1}{n_{1}}.$$

The total number of such sites will depend on $\theta \pi_{1}$, and in general on the factor $(\theta \pi_{1}{)}^{k_{1}}$ in (21) for larger numbers of latent mutations. But conditional on $k_{1}$, the site-frequency counts for a rare variant do not depend on θ, at least to leading order in the sample size *n*. If there are $k_{1}>1$ mutations in the ancestry of the rare variant, then $n_{1}$ cannot be less than $k_{1}$. This is shown in Fig. 2a for $k_{1}=2$ to $k_{1}=5$. A key effect of recurrent mutation is to give relatively less weight to low site-frequency counts, as found previously by Jenkins and Song (2011).

Using (21) and (23) the joint distribution of $n_{1}$ and $k_{1}$ obeys


(25) $$p(n_{1},k_{1};n\;\mathrm{large})\propto \frac{\left|S_{n_{1}}^{\left(k_{1}\right)}\right|(\theta \pi_{1}{)}^{k_{1}}}{n_{1}!}$$

which can be compared to the results of Jenkins and Song (2011). With fixed $n_{1}$ and large *n* in our model, all mutations in the ancestry of the rare variant will be non-nested mutations; note this also follows from (18) in Jenkins and Song (2011). Adapting the notation of Jenkins and Song (2011) in which $E_{2N,N}^{\left(1,1\right)}$ is the event that the $n_{1}$ copies of allele 1 are due to two non-nested mutations, both from allele K=2 to allele 1, their (21) becomes


$$p(n_{1},n_{2},E_{2N,N}^{\left(1,1\right)})\approx \theta^{2}\pi_{1}^{2}\frac{\left|S_{n_{1}}^{\left(2\right)}\right|}{n_{1}!}$$

for large $n∼n_{2}$ (and small θ), which is identical to (25) if $k_{1}=2$.

Numerical computations (not shown) using the unnumbered equation below (10) in Jenkins and Song (2011), which holds for any θ, reproduce the case of $k_{1}=2$ shown in Fig. 2a when *n* is large. This is evident in Figure 3 of Jenkins and Song (2011) for the quantity $E_{2NN}$. These computations are difficult for samples beyond the hundreds. Our results for $k_{1}=3$ could potentially also be compared to the $O(\theta^{3})$ results of Bhaskar *et al.* (2012) using their Theorem 3 and summing appropriately.

Figure 2b shows how the site-frequency counts of the rare variant depend on the mutation parameter of that variant, $\theta \pi_{1}$. Although Fig. 2a shows a dramatic effect of $k_{1}$ on the site-frequency counts, Fig. 2b suggests that large values of $k_{1}$ are unlikely. This is evident from (21) and (25) in that each additional mutation results in an additional factor of $\theta \pi_{1}$. Note that the smallest value of $\theta \pi_{1}$ in Fig. 2b is already more than twice the human average. From (23), we have


$$p(n_{1};n\;\mathrm{large},\theta \;\mathrm{small})\propto \frac{\theta \pi_{1}}{n_{1}}$$

which is consistent with (9) in the case where allele 1 is rare in a large sample. Thus, when $\theta \pi_{1}$ is small (0.002 and 0.02 in Fig. 2b) the site-frequency spectrum under recurrent mutation is very close to the standard infinite-sites model predictions. When $\theta \pi_{1}$ is large (0.2 in Fig. 2b) the site-frequency spectrum under recurrent mutation is noticeably different, with a dearth of low-frequency variants and corresponding excesses at higher frequencies. Figure 2b plots site frequencies on a log scale to better illustrate differences, especially at higher frequencies.

## Theory for nonconstant populations

Here we extend our analysis to populations which deviate from the standard neutral site-frequency predictions. We have in mind populations which have changed in size, although other applications may be possible. Here gene genealogies are the general coalescent trees of Griffiths and Tavaré (1998), which have the same branching structure of standard coalescent trees but may have different distributions of coalescence times.

Equation (21) suggests another way to model both the number of copies ($n_{1}$) of a variant of interest and the corresponding count of latent mutations ($k_{1}$) when the variant is rare in a large sample. Arratia *et al.* (1992) proved that when the sample size tends to infinity, the numbers of alleles in small counts 1,2,…,i in the Ewens distribution converge to independent Poisson random variables with expected values θ,θ/2,…,θ/i. Note that θ/i is the usual expected site-frequency count of mutants in *i* copies in the sample under the standard neutral model of a large constant-size population. A seminal result of Watterson (1974b) is that the numbers and counts of mutations in a sample from such a multi-type Poisson distribution conform to the Ewens sampling formula when conditioned on their total size. So we may interpret (21) and other findings in the previous section within this independent-Poissons sampling framework.

This is exactly the approach in the Supplementary Materials of Seplyarskiy *et al.* (2021). Again, human SNP data strongly reject the standard neutral model with site-frequencies ∝1/i, owing largely to the great excess of singletons and other rare variants due to our recent growth (Keinan and Clark 2012; Gazave *et al.* 2014). So we replace 1/i with $E[\tau_{i}]/2$, where $\tau_{i}$ is the total length of branches with *i* descendants in the gene genealogy of a sample. For an extension of independent-Poissons sampling to variants under selection, see Desai and Plotkin (2008). Our notation is different than in Seplyarskiy *et al.* (2021) because here we use the coalescent or diffusion time scale.

Under the standard neutral coalescent model, $E[\tau_{i}]=2/i$. For the general coalescent trees of Griffiths and Tavaré (1998), $\tau_{i}$ can be expressed in terms of the coalescent intervals, $T_{k}$, which are the lengths of time when there were k∈{2,…,n} lineages in the ancestry of the sample. In particular,


(26) $$E[\tau_{i}]=\sum_{k=2}^{n}kE[T_{k}]\frac{\left(\begin{array}{c} n-i-1\\ k-2 \end{array}\right)}{\left(\begin{array}{c} n-1\\ k-1 \end{array}\right)}$$

(Fu 1995; Griffiths and Tavaré 1998).

Watterson (1974b) studied three models. In Model 1, using our notation, mutations arise from a constant source at rate θ, then propagate or go extinct independently according to a critical branching process, i.e. with birth rate equal to death rate as for a neutral mutation. The number of mutations in count *i* has expected value $\theta \mu^{i}/i$, for a constant μ>0 which converges to 1 as the duration of the process increases. Watterson (1974b) proved that the numbers and counts of mutations follow the Ewens sampling formula when conditioned on their total size, which for Watterson (1974b) was equivalent to the population size. Models 2 and 3 are the Moran model and the Wright-Fisher model (Fisher 1930b; Wright 1931; Moran 1958, 1962) and Watterson (1974b) proved that these have the same limit as Model 1 when the population size is large.

Model 1 is an example of a logarithmic species distribution (Fisher 1943; Watterson 1974a; Arratia *et al.* 2003; Lambert 2011). Branching-processes have also been used to describe and infer the ages of rare alleles (Rannala and Slatkin 1997; Slatkin and Rannala 2000; Wiuf 2000); for recent developments and a review, see Crespo *et al.* (2021). Slatkin (2000) used this approach and an extension of Griffiths and Tavaré (1998) to model the ages of rare alleles in a large sample. Champagnat and Lambert (2012, 2013) studied the convergence of population frequencies of alleles for supercritical, subcritical or critical branching processes. All of these works assume that each allele traces back to a single mutation, as under the infinite-alleles mutation model.

Our approach to modeling recurrent mutation follows that of Watterson (1974b) to Model 1. Whereas Watterson (1974b) did not specify the source of mutations, here we take it to be the production of rare variants by mutation from a common variant on the gene genealogy of a large sample. What for Watterson (1974b) was the total population size is for us the total count of a rare variant. Allele 1 is our nominal variant of interest, but for simplicity for the moment, we use *n*, *k* and θ in place of $n_{1}$, $k_{1}$ and $\theta \pi_{1}$. As a further notational convenience, we define


$${\bar{\tau }}_{i}\equiv E[\tau_{i}]$$

so that $\theta \;{\bar{\tau }}_{i}/2$ is the expected number of mutations with count *i* in this independent-Poissons sampling model.

Let $\left(a_{1},a_{2},\ldots \right)$ be the numbers of latent mutations of the variant of interest with counts (1,2,…). We assume that $a_{i}∼\mathrm{Poisson}(\theta \;{\bar{\tau }}_{i}/2)$ and that $a_{i}$ and $a_{j}$ are independent for i≠j. Their joint distribution is then


(27) $$\begin{array}{rl} P(a_{1},a_{2},\ldots ) & =\prod_{i\geq 1}\frac{(\theta \;{\bar{\tau }}_{i}/2{)}^{a_{i}}}{a_{i}!}e^{-\theta \;{\bar{\tau }}_{i}/2}\\ & =e^{-\frac{\theta }{2}\sum_{i}{\bar{\tau }}_{i}}\prod_{i\geq 1}\frac{(\theta \;{\bar{\tau }}_{i}/2{)}^{a_{i}}}{a_{i}!} \end{array}$$

with $a_{i}\geq 0$. The total sample size is what would set the upper limits of the product and the sum above, but we leave these unspecified for now, only imagining that the total sample size is much larger than the sample count of the variant of interest, so we can model the latter without restriction.

We are only concerned with $a_{i}$ for i≤b, where *b* is the largest rare-variant count. Thus, the assumption of independence in (27), which is equivalent to there being no nested mutations in the ancestry of a rare variant, will only need to be true for ${\bar{\tau }}_{i}$ with i∈(1,…,b). In Appendix section “Low-count branches of general coalescent trees” we prove that this holds for the trees of Griffiths and Tavaré (1998) for fixed *b* in the limit as the total sample size tends to infinity, and that the counts $\left(a_{1},\ldots ,a_{b}\right)$ converge to independent Poisson random variables as with expected values $\left(\theta \;{\bar{\tau }}_{1}/2,\ldots ,\theta \;{\bar{\tau }}_{b}/2\right)$. A condition is that the total height of the genealogy is finite, which is a mild assumption ruling out pathological situations such as a populations whose sizes *increase* too quickly backward in time.

The count of the variant of interest is $n=\sum_{i}ia_{i}$ and its number of latent mutations is $k=\sum_{i}a_{i}$. Following Watterson (1974b), we consider the probability generating function of *n* and *k*, which in the present case simplifies to


$$\begin{array}{rl} G_{n,k}(x,y) & =\sum_{\left(a_{1},a_{2},\ldots \right)}P(a_{1},a_{2},\ldots )x^{n}y^{k}=e^{-\frac{\theta }{2}\sum_{i}{\bar{\tau }}_{i}}\sum_{k=0}^{\infty }\frac{(\frac{\theta }{2}{)}^{k}y^{k}}{k!}{\left(\sum_{i}x^{i}{\bar{\tau }}_{i}\right)}^{k}. \end{array}$$

For the details of this derivation, see (A29) in the Appendix. The coefficient of $x^{n}$ (and $y^{k}$) can be found using


(28) $${\left(\sum_{i}x^{i}{\bar{\tau }}_{i}\right)}^{k}=\sum_{n\geq k}x^{n}\sum_{\left(i_{1},\ldots ,i_{k-1}\right)}{\bar{\tau }}_{i_{1}}{\bar{\tau }}_{i_{2}}\cdots {\bar{\tau }}_{i_{k}}$$

where the sum is over


$$i_{m}=1,\ldots ,n-(k-m)-\sum_{g=1}^{m-1}i_{g}$$

for m=1,…,k−1, and with


$$i_{k}=n-\sum_{m=1}^{k-1}i_{m}.$$

Returning to our notation in which $n_{1}$ is the number of copies of a variant of interest, $k_{1}$ its number of latent mutations, $\theta \pi_{1}$ its mutation parameter, and *n* is the total sample size, and further using τ to show the new dependence on the vector of expected times $\left({\bar{\tau }}_{1},\ldots ,{\bar{\tau }}_{n-1}\right)$, we have


(29) $$p(n_{1},k_{1};n\;\mathrm{large},\tau )\approx \frac{{\left(\frac{\theta \pi_{1}}{2}\right)}^{k_{1}}\sum_{\left(i_{1},\ldots ,i_{k_{1}-1}\right)}\prod_{m=1}^{k_{1}}{\bar{\tau }}_{i_{m}}}{k_{1}!}e^{-\frac{\theta \pi_{1}}{2}\sum_{i=1}^{n-1}{\bar{\tau }}_{i}}$$

which is nonzero for $n_{1}=k_{1}=0$ and $n_{1}\geq k_{1}\geq 1$. The sum over $\left(i_{1},\ldots ,i_{k_{1}-1}\right)$ here is the same as in (28). It is equivalent to summing over partitions of the integers 1 through $n_{1}$ into $k_{1}$ subsets, where the sizes of the subsets are $\left(i_{1},\ldots ,i_{k_{1}}\right)$.

It is convenient to decompose (29) as follows. The number of type-1 mutations is Poisson distributed


(30) $$p(k_{1};n\;\mathrm{large},\tau )\approx \frac{{\left(\frac{\theta \pi_{1}}{2}\sum_{i=1}^{n-1}{\bar{\tau }}_{i}\right)}^{k_{1}}}{k_{1}!}e^{-\frac{\theta \pi_{1}}{2}\sum_{i=1}^{n-1}{\bar{\tau }}_{i}},$$

with parameter equal to the expected number of type-1 mutations on the gene genealogy of the sample. Conditional on this, the distribution of the number of times allele 1 appears in the sample is given by


(31) $$p(n_{1}|k_{1};n\;\mathrm{large},\tau )\approx \sum_{\left(i_{1},\ldots ,i_{k_{1}-1}\right)}\prod_{m=1}^{k_{1}}\frac{{\bar{\tau }}_{i_{m}}}{\sum_{i=1}^{n-1}{\bar{\tau }}_{i}},$$

which depends on the relative expected branch lengths but does not depend on θ or $\pi_{1}$.

Alternatively, $p(n_{1};n\;\mathrm{large},\tau )$ can be computed by summing (29) appropriately, over $k_{1}\in (0,\ldots ,n_{1})$. Then


(32) $$p(k_{1}|n_{1};n\;\mathrm{large},\tau )\approx \frac{\;p(n_{1},k_{1};n\;\mathrm{large},\tau )}{p(n_{1};n\;\mathrm{large},\tau )}$$

can be used to estimate the number of independent mutations which produced the observed copies a rare allele.

The sum over $\left(i_{1},\ldots ,i_{k_{1}-1}\right)$ in (31) and (29) is straightforward to evaluate but will become impractical if $n_{1}$ and $k_{1}$ become too large. In what follows, we consider $k_{1}\leq 7$ mutations at each site. Equation (30) suggests that this will be accurate up to about three expected mutations per site, because the probability of $k_{1}$ greater than 7 is just over 1% when $(\theta \pi_{1}/2)\sum_{i=1}^{n-1}{\bar{\tau }}_{i}=3$. As in Fig. 2, the largest value of $n_{1}$ we consider is 40. These are not the upper limits of feasibility; it takes two minutes to evaluate (31) for all $k_{1}\in \{0,\ldots ,7\}$ and $n_{1}\in \{0,\ldots ,40\}$ in Mathematica version 11.2 (Wolfram Research, Inc. 2017) on a mid-2015 MacBook Pro.

Considering the first three possible values of $k_{1}$ in (31),


(33) $$\begin{array}{rl} \;p(n_{1}|0;n\;\mathrm{large},\tau ) & \approx \left\{ \begin{array}{cc} 1 & \mathrm{if}\;n_{1}=0\\ 0 & \mathrm{if}\;n_{1}\geq 1 \end{array}\right. \end{array}$$

(34) $$p(n_{1}|1;n\;\mathrm{large},\tau )\approx \frac{{\bar{\tau }}_{n_{1}}}{\sum_{i=1}^{n-1}{\bar{\tau }}_{i}}$$

(35) $$p(n_{1}|2;n\;\mathrm{large},\tau )\approx \frac{\sum_{i=1}^{n_{1}-1}{\bar{\tau }}_{i}{\bar{\tau }}_{n_{1}-i}}{{\left(\sum_{i=1}^{n-1}{\bar{\tau }}_{i}\right)}^{2}}$$

Equation (33) says simply that if there are no type-1 mutations on the gene genealogy then no copies of allele-1 will be observed. Equation (34) is the familiar result for the site-frequency spectrum, that it is given by the proportion of branches in the tree that have $n_{1}$ descendants. Equation (35) extends this to two mutations and emphasizes that mutations in the ancestry of a rare allele will be non-nested when *n* is large.

For the constant-size model, we find new approximations


(36) $$p(n_{1};n\;\mathrm{large},{\bar{\tau }}_{i}=2/i)\approx \frac{(\theta \pi_{1}{)}^{\left(n_{1}\right)}}{n_{1}!}e^{-\theta \pi_{1}\sum_{i=1}^{n-1}1/i}$$

(37) $$p(n_{1}|k_{1};n\;\mathrm{large},{\bar{\tau }}_{i}=2/i)\approx \frac{\left|S_{n_{1}}^{\left(k_{1}\right)}\right|k_{1}!}{n_{1}!}{\left(\sum_{i=1}^{n-1}\frac{1}{i}\right)}^{-k_{1}}$$

(38) $$p(n_{1},k_{1};n\;\mathrm{large},{\bar{\tau }}_{i}=2/i)\approx \frac{\left|S_{n_{1}}^{\left(k_{1}\right)}\right|(\theta \pi_{1}{)}^{k_{1}}}{n_{1}!}e^{-\theta \pi_{1}\sum_{i=1}^{n-1}1/i}$$

corresponding to (23), (24) and (25), respectively, in which the condition ${\bar{\tau }}_{i}=2/i$ should be taken to hold for all i∈{2,…,n}. Figure 2 is unchanged if (36) and (37) are used instead of (23) and (24). Also, the conditional probability of $k_{1}$ given $n_{1}$ from (36) and (38) is identical to (21).

### Relation to *K*-alleles diffusion results

From a gene-genealogical point of view, (36) is the probability of seeing $n_{1}$ total copies of a rare variant when a random number of type-1 mutations occurs on the low-count branches of a standard neutral coalescent tree. However, the type of the common variant and the ancestral states of these mutations are not specified in the independent-Poissons model. Of course these should be allele *K*, as in “A conditional ancestral process for rare variants,” but (36) does not include this event. In contrast, the sampling probabilities (3) and (5) from the equilibrium diffusion model specify the types of the entire sample. Implicitly, they average over the ancestral states of the sample. Here we focus on K=2 and show how (36) is related to (5) when *n* is large, in particular to the leading order term in the expansion (22).

The type of the common ancestor of the entire sample, at the root of the coalescent tree, is allele 2 with probability $\pi_{2}$. If this were the case, allele 2 would be the ancestral source of the low-count type-1 mutations. But if θ is not very small, it is possible for allele 2 to be the ancestral source of these mutations even if the common ancestor is type 1. To illustrate, dividing either (5) or (22) by (36) and letting n→∞ gives


(39) $$\frac{e^{\theta \pi_{1}\gamma }\Gamma (\theta )}{\Gamma (\theta \pi_{2})}=\pi_{2}+O(\theta^{2}).$$

Indeed when θ is small, (22) is close to (36) times $\pi_{2}$. But the error of this, even as *n* tends to infinity, may be appreciable for larger values of θ. The additional probability of order $\theta^{2}$ in (39) is consistent with the possibility that the root of the coalescent tree is type 1 and there are two type-2 mutations, one on each of the two branches descending from the root.

A better guarantee that allele 2 is the ancestral source of low-count mutations would be to specify it not as type of the single most recent common ancestor but rather as the type of the pair of ancestors at the first time in the past when there were two ancestral lineages. Equation (5), with sample size equal to two, gives the relevant probability. This accounts for both possible states at the root of the tree as well as for mutation during the deepest coalescent interval, $T_{2}$ in (26). Then the independent-Poissons model could be applied to the remainder of the tree, i.e. to coalescent intervals $T_{3}$ through $T_{n}$.

Because latent mutations of rare variants tend to be very recent, cf. (18) and (19), we may extend this logic to the first time in the past when there were *r* ancestral lines of the sample, for an arbitrary r≥1. The probability that these are all of type 2 is given by the diffusion result (5) with sample size *r*. The probability of seeing $n_{1}$ copies of the rare variant is given by an appropriately adjusted independent-Poissons model, covering coalescent intervals $T_{r+1}$ through $T_{n}$. By summing (26) only over j∈{r+1,…,n} it can be shown that the total length of branches with *i* descendants in this more recent part of the gene genealogy differs only by $2(1-r)/n+O(1/n^{2})$ from the full result ${\bar{\tau }}_{i}=2/i$. The product of these two probabilities is


(40) $$\frac{\Gamma (\theta )\Gamma (\theta \pi_{2}+r)}{\Gamma (\theta \pi_{2})\Gamma (\theta +r)}\frac{(\theta \pi_{1}{)}_{n_{1}}}{n_{1}!}e^{-\theta \pi_{1}\sum_{i=r}^{n-1}1/i}$$

which can be compared to the leading order term in (22).

As expected from (39), if r=1 (40) reduces to (36) times $\pi_{2}$. Now dividing (5) or (22) by (40) and letting n→∞ gives


(41) $$\frac{\Gamma (\theta \pi_{2}+r)}{\Gamma (\theta +r)}e^{-\theta \pi_{1}\left(\sum_{i=1}^{r-1}1/i-\gamma \right)}$$

as a measure of how well this augmented independent-Poissions model approximates the equilibrium diffusion result, depending on *r* and θ. Expanding (41) around θ=0, because we do not in fact expect the per-site mutation parameter to be large, gives


(42) $$1+\frac{(2-\pi_{1})\pi_{1}}{2}\left(\frac{\pi^{2}}{6}-\sum_{\;j=1}^{r-1}\frac{1}{j^{2}}\right)\theta^{2}+O\left(\theta^{3}\right)$$

where the π in $\pi^{2}$ is the usual constant (not our $\pi_{i}$). The parenthetic term in (42) tends to zero quickly as *r* increases. It is equal to the trigamma function $\psi^{\left(1\right)}(r)$ for r∈{1,2,3,…}; see 6.4.2 and 6.4.3 in Abramowitz and Stegun (1964). Even just taking r=2 instead of r=1 cuts the error by about 60%.

Similar conclusions may be drawn from the large-*r* expansion of (41), which gives $1+(2-\pi_{1})\pi_{1}\theta^{2}/(2r)+O(1/r^{2})$. Again $\theta^{2}$ is the largest-order effect of mutation. The event that a pair of mutations occurs on the two lineages descending from the root of the coalescent tree is non-negligible in the constant-size population model, even as n→∞ and even for the entire population, because ancient coalescence times tend to be long. But the chance of this event will be small for most eukaryote species as θ ranges from about $10^{-4}$ to $10^{-1}$ with typical values around $10^{-2}$ (Leffler *et al.* 2012). Based on our estimates in the next section, even the fastest-mutating sites in the human genome have θ≈0.02. Note that this event will even less likely in growing populations, because in this case the deepest coalescence times will be relatively short, but could be an important phenomenon for populations which were much larger in the past.

## Theoretical example and data application

Here we illustrate the theoretical and empirical use of (30) and (31). First we describe the consequences of recurrent mutation in an exponentially growing population compared to those in a population of constant size. Second we explore an entirely empirical application to human SNP data, which suggests that disparate site-frequency spectra may be explained by differences in mutation rate (and thus recurrent mutation).

Note that if estimates of the expected fraction of the gene genealogy comprised of branches with *i* descendants, that is


(43) $$\frac{{\bar{\tau }}_{i}}{\sum_{i=1}^{n-1}{\bar{\tau }}_{i}}=\frac{E[\tau_{i}]}{\sum_{i=1}^{n-1}E[\tau_{i}]},$$

are available, then $p(n_{1}|k_{1};n\;\mathrm{large},\tau )$ can be computed using (31). In addition, for any estimated or supposed values of the expected number of mutations on the gene genealogy,


(44) $$\frac{\theta \pi_{1}}{2}\sum_{i=1}^{n-1}{\bar{\tau }}_{i}=\frac{\theta \pi_{1}}{2}\sum_{i=1}^{n-1}E[\tau_{i}],$$

the joint distribution of the number of latent mutations, $k_{1}$, and their total count, $n_{1}$, is the product of (30) and (31).

### An exponentially growing population

Consider the simple model of pure exponential growth which has been the subject of a number of studies (Slatkin and Hudson 1991; Griffiths and Tavaré 1998; Polanski and Kimmel 2003; Chen and Chen 2013; Polanski *et al.* 2017): a population which has reached its current (haploid) size $N_{0}$ by exponential growth at rate *r* per generation. On the coalescent time scale of $N_{0}$ generations, looking backward in time and setting $\beta =N_{0}r$,


(45) $$N(t)=N_{0}e^{-\beta t}$$

gives the population size at time *t* in the past. This model is unrealistic because the past population size approaches zero, but it can be taken as a rough approximation for recent dramatic growth. For instance, a population of current size $N_{0}=5\times 10^{7}$ with a generation time of 30 years and r=0.0064, would have $\beta =3.2\times 10^{5}$. About 40,000 years ago, it would have had size $10^{5}$, and using equation (7) in Slatkin and Hudson (1991) the pairwise coalescence time would be about 57,000 years.

The expectation $E[\tau_{i}]$ can be computed from (26) if the expected coalescent intervals $E[T_{k}]$ are known. We use the large-*n* results of Chen and Chen (2013) for $E[T_{k}]$ (our notation) to obtain a simple approximation for $E[\tau_{i}]$. With the time scale and notation here, equation (11) in Chen and Chen (2013) gives


(46) $$\frac{1}{\beta }\log\;\left(2\beta \left(\frac{1}{k}-\frac{1}{n}\right)+1\right)$$

as a large-*n* approximation for the cumulative expected time for the number of ancestral lineages of the sample to decrease from *n* to *k*. Writing (46) as a continuous function of x=k/n,


(47) $$f(x)=\frac{1}{\beta }\log\;\left(\frac{2\beta }{n}\frac{1-x}{x}+1\right),$$

we approximate the expected coalescent interval as


(48) $$\begin{array}{rl} E[T_{k}] & =f(x-dx)-f(x)\approx -f^{\prime }(x)\;dx\\ & =\frac{2}{x(2\beta (1-x)+xn)}. \end{array}$$

Note that while (48) is a large-*n* approximation, it allows that β might be of the same order of magnitude as *n*. Applying the same approximation to the combinatorial coefficient in (26) gives


(49) $$\frac{\left(\begin{array}{c} n-i-1\\ k-2 \end{array}\right)}{\left(\begin{array}{c} n-1\\ k-1 \end{array}\right)}\approx \frac{x}{1-x}{\left(1-x\right)}^{i}.$$

Finally, we approximate the sum in (26) with the integral


(50) $$\begin{array}{rl} E[\tau_{i}] & \approx \int_{0}^{1}xn\frac{2}{x(2\beta (1-x)+xn)}\frac{x}{1-x}(1-x{)}^{i}\;dx,\\ & =\frac{n}{\beta }\int_{0}^{1}{\left[1-\left(1-\frac{n}{2\beta }\right)x\right]}^{-1}x(1-x{)}^{i-1}dy \end{array}$$

(51) =nβi(i+1)F12(1,2;i+2;1−n2β)

which can be evaluated efficiently either as (51), in terms of the hypergeometric function, or as the integral (50). Slatkin and Hudson (1991) and others have observed that gene genealogies under very fast exponential growth are close to star trees. Using either (50) or (51) we have


(52) $$E[\tau_{i}]\approx \left\{ \begin{array}{cc} \frac{\log\;(2\beta /n)-1}{\beta /n} & \mathrm{if}\;i=1\\ \frac{1}{i(i-1)\beta /n} & \mathrm{if}\;i\geq 2 \end{array}\right.$$

as β/n increases. From the log(2β/n) term in (52), we confirm the star-tree prediction that under extreme growth essentially all variants will be singletons.

These results for exponentially growing populations, derived here using a coalescent approach, are identical in form to some results for “Luria-Delbrück distributions,” especially in application to cancer, derived using forward-time birth-death or branching processes (Luria and Delbrück 1943; Lea and Coulson 1949; Durrett 2013, 2015; Kessler and Levine 2013; Ohtsuki and Innan 2017; Cheek and Antal 2018; Gunnarsson *et al.* 2021; Poon *et al.* 2021). In particular, (50) has the same form as the approximation in equation (4) of Ohtsuki and Innan (2017) and as equation (33) in Gunnarsson *et al.* (2021). Equation (52) has the same form as the expression in Theorem 2 in Durrett (2013) if only the leading-order term is kept in (52) in the case i=1.

**Fig. 3.**
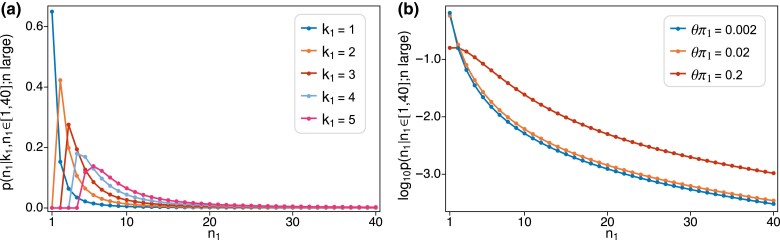
Plots of the same quantities shown in Fig. 2 but for a sample of size $n=10^{5}$ under pure exponential growth with β/n=3. a) Probability of observing $n_{1}$ copies of allele 1 in the sample given $k_{1}=1,2,3,4,5$ latent mutations. b) $\log_{10}$-probability of observing $n_{1}$ copies of allele 1 in the sample for three different mutation rates, corresponding to the values of $\theta \pi_{1}$: 0.002, 0.02 and 0.2 in Fig. 2, but here expressed in terms of expected numbers of mutations on the gene genealogy (44): 0.024, 0.24 and 2.4. Probabilities in both panels are normalized to sum to one for $n_{1}\in \{1,2,\ldots ,40\}$.

Figure 3 shows the same quantities as Fig. 2 but for the pure exponential growth model with $n=10^{5}$ and β/n=3. The value β/n=3 was chosen to roughly reproduce the ratio of singletons to doubletons observed for low-rate sites in the gnomAD data in section “Application to human SNP data.” Figure 3a is directly comparable to Fig. 2a, the only difference being whether $E[\tau_{i}]=2/i$ or comes from (51). As Fig. 3a shows, recent rapid growth produces a single-mutation ($k_{1}=1$, blue line) site-frequncy spectrum with an excess of rare variants and a deficit of common variants. So, compared to the constant-size case in Fig. 2a, there is a diminished tendency to observe high-frequency variants when the number of latent mutations is larger, and a stronger tendency for the site-frequency count ($n_{1}$) to be equal to or close to the number of latent mutations.

To make Fig. 3b comparable to Fig. 2b, we used (44) with $n=10^{5}$ and $E[\tau_{i}]=2/i$ to compute the corresponding expected numbers of mutations on the gene genealogy for the three values of $\theta \pi_{1}$ in Fig. 2  (0.002,0.02,0.2). The resulting expected numbers of mutations were 0.024, 0.24 and 2.4, the last being about equal to the value for the highest-rate sites in the gnomAD data in section “Application to human SNP data.” We then computed $p(n_{1};n\;\mathrm{large},\tau )$ by averaging (31) over the distribution (30). Similar to Fig. 2b, the two smaller values of the mutation rate give nearly indistinguishable results for the total count $n_{1}$. But there is a dramatic difference for the largest mutation rate. In Fig. 2b the prediction is distinctly L-shaped and thus similar to that for the lowest mutation rate, which again is 100-fold lower. In contrast, in Fig. 3b singletons have a much lower chance of being observed. In fact, doubletons are slightly more likely than singletons. This relative excess of doubletons is due to the fact when there are two latent mutations these are highly likely to produce two copies of the variant under growth (Fig. 3a) than under constant size (Fig. 2a).

**Fig. 4.**
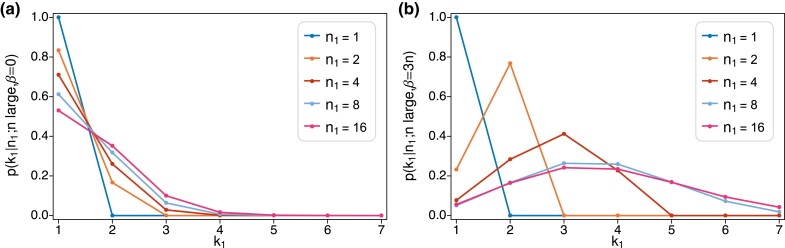
Probabilities of $k_{1}=1,2,3,4,5,6,7$ latent mutations for increasing values of $n_{1}$—1, 2, 4, 8, and 16—when 2.4 mutations are expected on the gene genealogy of a sample of size $n=10^{5}$ (or equivalently $\theta \pi_{1}=0.2$ in the constant-size case). Panel A plots (21) with $\theta \pi_{1}=0.2$. Panel B shows the same probability computed using (30) and (31) under exponential growth with β/n=3.

It is also of interest to know how the number of latent mutations in the ancestry of a rare variant depends on its count. Figure 4 depicts this for a series of increasing counts $n_{1}$, from 1 to 16. Figure 4a shows the results for constant size, Fig. 4b the corresponding results for pure exponential growth. The expected number of mutations on the gene genealogy is 2.4 in both cases. Regardless of the demography, if only one copy of the variant is observed, it must be due to one mutation. Otherwise, the results differ greatly for constant size versus growth. Under constant size, a variant observed multiple times in the sample can easily be due to a single mutation. Under growth, higher variant counts are more likely due to multiple mutations.

### Application to human SNP data

We also used (30) and (31) to account for latent mutations in the ancestry of rare variants in a subset of the gnomAD data (Karczewski *et al.* 2020). We took the approach described in the Supplementary Materials of Seplyarskiy *et al.* (2021), specifically obtaining estimates of relative branch lengths (43) from the data at low-rate sites, then using our new analytical result (31) to average over mutation counts. Rather than categorizing variants by trinucleotide context as in Seplyarskiy *et al.* (2021), we analyzed data from gnomAD version v2.1.1, presorted into 109 bins based on estimates of mutation rate by the Roulette method of Seplyarskiy *et al.* (2022) which incorporates information from the six flanking bases on either side of a SNP, strand asymmetry, expression level, methylation and promoter status. We did not use this information but simply assumed that variants within a bin all have the same mutation rate.

The data consist of variant counts for synonymous mutations in the exomes of about 57 K non-Finnish Europeans. Thus n∼114 K although this varied by about 2% among sites because we required that sites were successfully genotyped in a minimum of 112K chromosomes. Importantly for our application, the data include monomorphic sites, i.e. sites with variant count equal to zero. The gnomAD only provides *n* for polymorphic sites, so we imputed *n* for monomorphic sites using the nearest value at a polymorphic site within 100 bp on either side of the focal site. After filtering for sequencing quality and coverage as well as removing mutation rate bins with fewer than 100 observed mutations, there are a total of 12,338,176 sites in 97 bins and 834,486 of these are polymorphic.

**Fig. 5.**
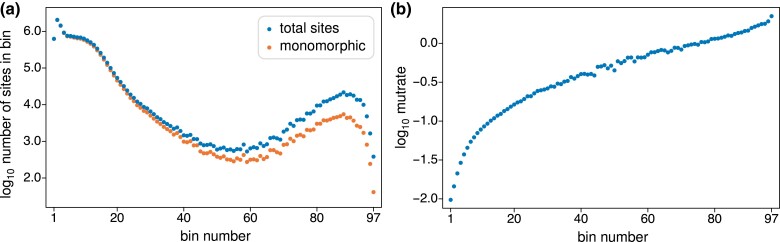
a) Total numbers of sites and total numbers of monomorphic, or invariant, sites in the gnomAD data for each of the 97 bins. b) Estimated mutation rates—i.e. the “mutrate” or expected number of latent mutations $(\theta \pi_{1}/2)\sum_{i}{\bar{\tau }}_{i}$ as discussed in the text—on a log scale for bins 1 through 97.

Figure 5a shows the total numbers of sites and the numbers of monomorphic sites in each bin. The great majority of sites are in bins 1 through roughly 20. These have low mutation rates, as indicated by their nearly equal numbers of total sites and monomorphic sites. The widening gap between the total number of sites and the number of monomorphic sites reflects the fact that higher-number bins have larger mutation rates.

For each bin, the data are the numbers of sites where a variant is observed in each possible count in the sample. As in “Latent mutations and sample counts of rare alleles,” these are marginal with respect to other possible variants at the site. Sites with two (resp. three) rare variants appear twice (resp. three times) in the data, once for each rare variant. These will likely be in different bins given the fine substructure of mutation rate variation (Seplyarskiy *et al.* 2021, 2022). Although bins contain mixtures of different sequence contexts and different nucleotide substitutions, for our purposes sites within a bin are all of the same type because they all have the same mutation rate.

Let $S_{i}$ be the number of sites in a given bin where *i* copies of the variant are observed in the sample. If a bin contains *L* total sites, then with reference to the notation in (2) we may write


(53) $$E[S_{i}]=LP[N_{1}=i;n],\;i\in \{0,\ldots ,n-1\}.$$

Thus we use a simplified notation here, with *i* in place of $n_{1}$ to avoid the additional subscript when we apply the results of the previous sections. In addition we use “mutrate” to refer to the estimate of the expected number of latent mutations per site for a given bin, i.e. $(\theta \pi_{1}/2)\sum_{i}{\bar{\tau }}_{i}$ for sites in that bin, as this is the rate parameter in the Poisson distribution (30).

We used (30) and the proportion of monomorphic sites, $S_{0}/L$, to estimate this “mutrate” for each bin, specifically as $-\log\;(S_{0}/L)$. Figure 5b plots these estimates across bins, on a log scale. They range from 0.0097 for bin 1 to 2.23 for bin 97, with a mean of 0.083, taking the proportion of sites in each bin into account. Most sites have mutation rates on the lower side: bins 1 through 5 contain about 47% of all sites, bins 1 through 19 about 95%, and bins 60 through 97 contain only about 2% of sites. Overall, rates vary 230-fold from lowest to highest. Assuming that the average estimated mutrate of 0.083 corresponds to the genome average mutation rate per site, for which the usual estimate of θ from pairwise differences is about 1/1300∼0.00077, we can infer that the expected number of mutations between a pair of (haploid) genomes is about $9\times 10^{-5}$ for the slowest sites and about 0.02 for the fastest sites.

We compared observed and expected site-frequency counts for each bin based on an empirical fit of our model. First, we used (30) with the estimated mutrate $(\theta \pi_{1}/2)\sum_{i}{\bar{\tau }}_{i}$ for each bin to compute probabilities of k∈{0,1,…,7} latent mutations. Then from (34) and the fact the polymorphisms at sites with very low mutation rates likely have just one latent mutation, we used the combined data for bins 1 through 5 to estimate ${\bar{\tau }}_{i}/\sum_{i}{\bar{\tau }}_{i}$ directly as $S_{i}/(L-S_{0})$ for i∈{1,…,40}. Our estimates of the mutrate for bins 1-5 range from 0.0097 to 0.037 with an average of 0.021, which we note is somewhat less than the smallest mutation rate in Figures 2 and 3. We assumed that this ${\bar{\tau }}_{i}/\sum_{i}{\bar{\tau }}_{i}$ estimated from bins 1–5 holds for all bins. Finally, we computed the expectations $E[S_{i}]$, for i∈{0,…,40} in each bin, multiplying the probabilities of counts obtained using (30) and (31) by the total number of sites in the bin, cf. (53).

**Fig. 6.**
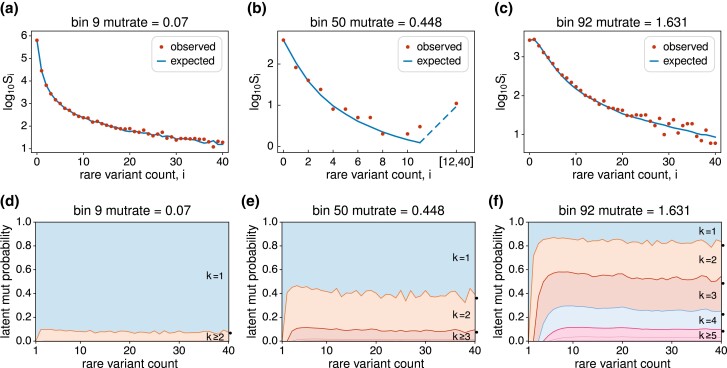
Upper three panels: Examples of model fit for a) a low-rate bin, b) a middle-rate bin, and c) a high-rate bin. Lower three panels: Stacked probabilities of k∈{1,2,3,4,5,6,7} latent mutations for rare variants with counts i∈{1,2,…,20} for the same three bins. As in Fig. 5, “mutrate” indicates an estimate of the expected number of latent mutations per site, $(\theta \pi_{1}/2)\sum_{i}{\bar{\tau }}_{i}$. Black dots on the right in the lower three panels show the probabilities for the shifted-Poisson result discussed in the text.

**Fig. A2.**
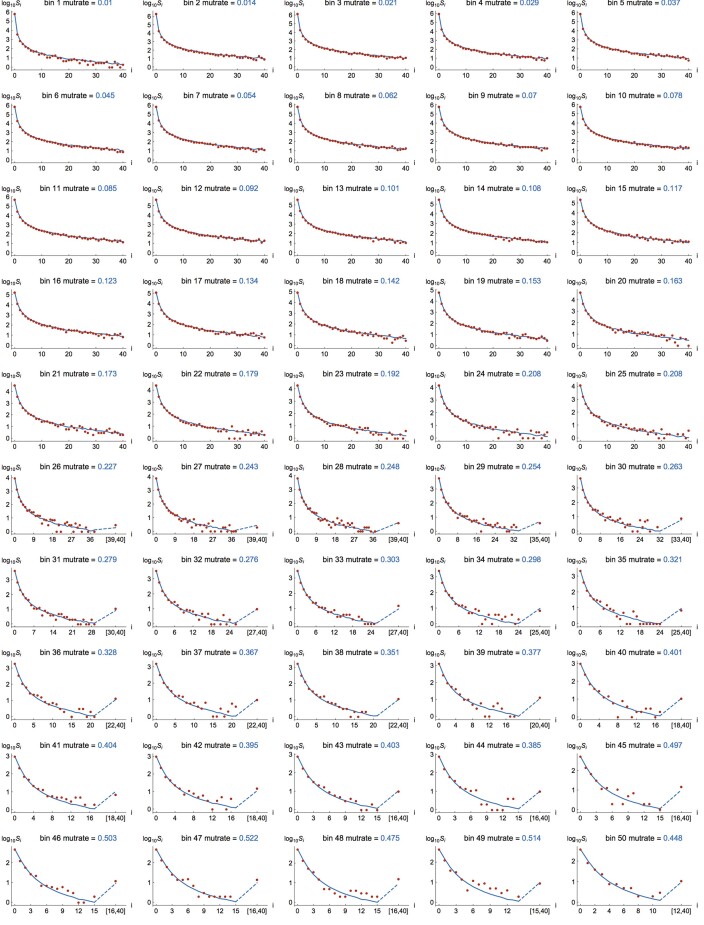

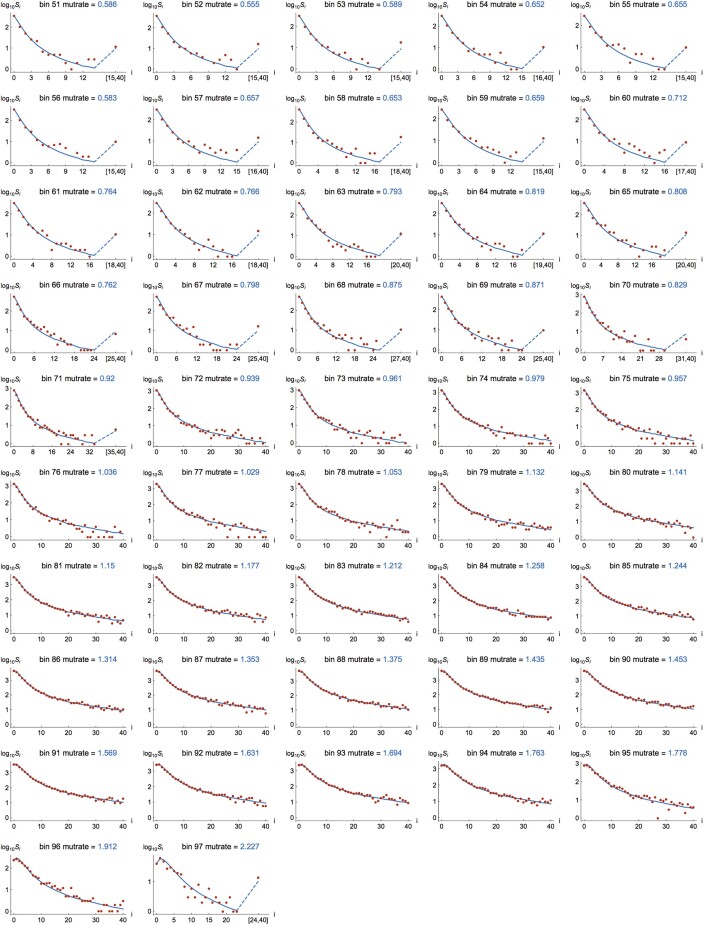
Plots like those in Fig. 6 for each of the 97 mutation-rate bins. (continues on next page)

The upper three panels of Fig. 6 show the observed and expected variant counts, $S_{i}$ for i∈{1,…,40}, for bins 9, 50 and 92, chosen to represent a low-rate bin, a middle-rate bin and a high-rate bin. Figure A2 in the Appendix gives the plots for all 97 bins. In making these plots, we grouped variant counts for which $E[S_{i}]<1$. For bin 50 for example, this was true of variant counts i∈[12,40] as depicted in Fig. 6B and in the 50th panel of Fig. A2. The mutrate values in these plots are again the estimates of the expected number of mutations per site on the gene genealogy, $(\theta \pi_{1}/2)\sum_{i}{\bar{\tau }}_{i}$, for each bin.

The broad pattern from these plots is clear. For smaller mutation rates (e.g. Fig. 6a) the site-frequency spectrum is heavily weighted toward the rarest variants. For large mutation rates (e.g. Fig. 6c), that is when multiple latent mutations are likely, the site-frequency spectrum is shifted toward higher counts. Again from Fig. 5a, the data contain fewer sites with intermediate mutation rates. In this case (e.g. Fig. 6b), the site-frequency spectrum does show the expected intermediate pattern, but subject to considerable sampling error. Across the range of mutation rates, the empirical model, which uses low-rate sites to estimate relative branch lengths ${\bar{\tau }}_{i}/\sum_{i}{\bar{\tau }}_{i}$ and assumes these hold for all sites, fits the data well.

As can be seen in Fig. 6a and the first 20 or so panels of Fig. A2, the empirical estimates of ${\bar{\tau }}_{i}/\sum_{i}{\bar{\tau }}_{i}$ include fluctuations due to sampling error for higher-count variants. The combined data for the first five bins have $S_{i}$ ranging from 71 to 38 for i∈[30,40]. The presence of these fluctuations helps illustrate a subtler phenomenon, namely the smoothing which occurs at larger mutation rates (e.g. Fig. 6c). For reference, the combined data for the first five bins have $S_{i}$ in the thousands for the low-count variants. From these, the estimated chance that a latent mutation is a singleton is about 64%, followed by 13% for doubletons and 6% for tripletons. By comparison, the chance is less than 0.1% for each variant with count i∈[25,40]. The predictions $E[S_{i}]$ are smoothed for higher-count variants at larger mutation rates because they are mixtures. For example, two latent mutations will come in counts 1 and i−1, 2 and i−2, or 3 and i−3 with approximate relative proportions 64:13:6.

The lower three panels of Fig. 6 show estimates of the probability that a variant in count i∈{1,…,40} descends from k∈{1,…,7} latent mutations, computed using (32). All singletons descend from single mutations. Variants in larger counts can have multiple latent mutations, and the probabilities of these increase very quickly then settle down to stable values. This suggestion of a limiting distribution was also seen for exponential growth in Fig. 4b, only there depicted differently. For very large counts of the variant, the distribution of k−1 is well approximated by a Poisson with mean equal to the expected number of mutations per site on the gene genealogy, $(\theta \pi_{1}/2)\sum_{i}{\bar{\tau }}_{i}$. This shifted-Poisson result is known already for the constant-size case (Arratia *et al.* 2000; Yamato 2017). In “A remark on the total number of mutations for large $n_{1}$” in the Appendix we argue that it should hold more generally. The accuracy of this shifted-Poisson result for the gnomAD data and i=40 is shown by the black dots on the right axes of Figs. 6d–f.

For low-rate sites (e.g. Fig. 6d) there is a relatively small chance of multiple latent mutations. But the chance of two or more latent mutations is not negligible, owing to the very large sample size. Note that the mutrate for bin 9 is less than the genome average, which is 0.083 for this sample of n∼114K. Thus in a very large sample even low-rate sites are affected by recurrent mutation. For the middle-rate sites (e.g. Fig. 6e) in the trough in Fig. 5a the chance of there being only one latent mutation is still considerable. However, for high-rate sites (e.g. Fig. 6f) it can be more likely that there are two or three mutations in the ancestry of a rare variant than the single unique mutation which is typically supposed.

**Fig. 7.**
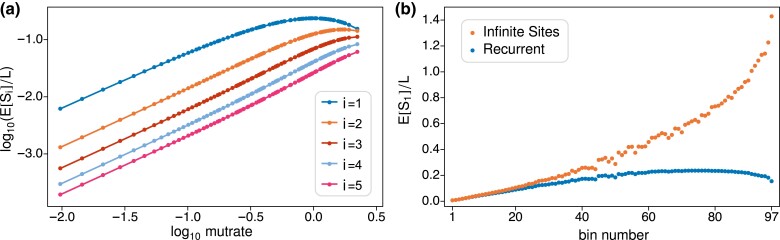
a) Predicted frequencies of rare variants as a function of mutrate across the 97 bins. $E[S_{i}]/L$ is the expected proportion of sites at which the rare variant is found in i∈{1,2,…,5} copies in the sample. The human genome average mutation rate is −1.08 on this scale. b) Predicted frequencies of singletons $E[S_{1}]/L$ in each bin under the infinite sites mutation model and under the independent-Poissons model of recurrent mutation.

Finally, we explored the extent to which rare variants might be observed less frequently than would be expected if there were no recurrent mutation. Figure 7a shows the expected frequency of singletons, doubletons, etc., up to variants found in five copies in the sample, across the range of mutrates in the binned gnomAD data. The standard infinite-sites prediction is that the frequency will increase linearly with the mutation rate. Figure 7a is largely consistent with this but shows marked deviations when the mutrate becomes too large. The point at which the linear prediction fails depends on the count of the rare variant. Singletons are the first to deviate, which they do as soon as there is an appreciable chance of two or more mutations at a site. For rare variants in five copies, linearity holds even close the upper limit of mutation rates in the human genome.

Figure 7b shows the extent to which the infinite-sites model over-predicts the frequency of singletons across the 97 bins. The infinite-sites prediction for a bin is its mutrate $(\theta \pi_{1}/2)\sum_{i}{\bar{\tau }}_{i}$ times the proportion of singleton branches ${\bar{\tau }}_{1}/\sum_{i}{\bar{\tau }}_{i}=0.64$ estimated from the first five bins. The corresponding independent-Poissons predictions are the same as those for i=1 in the 97 panels of Fig. A2. The infinite-sites model makes reasonable predictions for the twenty lowest-rate bins, which contain 96% of all sites and have mutation rates less than twice the genome average. But it predicts the impossible for the seven highest-rate bins: more singletons than there are sites to mutate. For bins 21 through 97, which contain 4% of all sites, the infinite-sites model predicts a total of 269,222 singletons compared to the 83,002 which are actually observed.

We emphasize that the results in Fig. 7 depend on the sample size. The expected number of mutations at a single site, $(\theta \pi_{1}/2)\sum_{i}{\bar{\tau }}_{i}$, is proportional to the total length of the gene genealogy, which is an increasing function of the sample size. Already for the sample size n∼114K considered here, singletons start to be affected by recurrent mutation at around the genome average mutation rate (Figs. 7 and 6d). For variants in any fixed count *i* there will be a sample size above which the infinite-sites, linear prediction starts to fail.

## Discussion

In this work, we modeled the mutational ancestry of a rare variant in a large sample. Under the standard neutral model of population genetics with *K*-allele parent-independent mutation, we found that co-segregating rare variants may be treated independently and that the Ewens sampling formula gives the probabilistic structure of latent mutations in their ancestries. In particular, the number of latent mutations is distributed like the number of alleles in the Ewens sampling formula. We obtained more general results, for changing population size, by modeling latent mutations as independent Poisson random variates.

Our aim was to describe how the site-frequency spectra of rare variants in large samples are affected by recurrent mutation. The key parameters for a variant in count *i* are its expected total rate of mutation on the gene genealogy of the sample (here denoted $(\theta \pi_{1}/2)\sum_{i}{\bar{\tau }}_{i}$ and called “mutrate” in the previous section) and the expected relative lengths of branches in the gene genealogy which have *i* descendants in the sample (${\bar{\tau }}_{i}/\sum_{i}{\bar{\tau }}_{i}$). Under the standard neutral model ${\bar{\tau }}_{i}=2/i$.

We obtained new results for ${\bar{\tau }}_{i}$ under exponential population growth and used these to illustrate how recurrent mutation affects the site-frequency spectrum differently than under constant size. Lastly, we showed that our general results provide a good fit to synonymous variation among a large number of (non-Finnish European) individuals in the human Genome Aggregation Database (Karczewski *et al.* 2020), suggesting that, whatever the causes of deviations from ${\bar{\tau }}_{i}=2/i$ might be for this sample, differences in mutation rate can explain differences in site-frequency spectra among sites.

Our application was empirical. We did not fit a demographic model, but following Seplyarskiy *et al.* (2021) used low-mutation-rate sites to estimate relative branch lengths and assumed these hold for all sites. Site-frequency spectra are a rich source of information about population-genetic phenomena but are of somewhat limited use in disentangling their effects (Myers *et al.* 2008; Bhaskar and Song 2014; Terhorst and Song 2015; Lapierre *et al.* 2017; Rosen *et al.* 2018). When low-mutation-rate sites are plentiful enough to provide stable estimates of relative branch lengths, this empirical method offers a way to control for myriad factors and isolate the effects of variation in mutation rate.

We began with a *K*-allele model with parent-independent mutation, and used its sampling probabilities in our computations for constant-size populations. We conjecture that our findings will hold for general mutation models because conditioning on a rare variant in a large sample means that the common allele will be the ancestral source of mutations with very high probability. Then the relevant mutation rate in any model will be the rate of the production of the rare allele from the common allele.

We described our general results as being for populations which may have changed in size. This is appropriate for the general coalescent model (Griffiths and Tavaré 1998) which we assumed for some proofs in the Appendix. Strictly speaking, the general coalescent does not require a generative model for the times between coalescent events. Thus our results can be applied more broadly. The case of a fixed tree with arbitrary $\tau_{i}$ considered in the Appendix is one example. The independent-Poissons model, with results (27) to (35), does not even require interpretation in terms of coalescence times. These results hold if we replace $\theta \pi_{1}{\bar{\tau }}_{i}/2$ with an arbitrary rate parameter $\lambda_{i}$ for the production of mutants in count *i*. Rates of production of mutants have been obtained for under a range of demographies and some types of selection (Lange and Fan 1997; Dorman *et al.* 2004; Lambert 2011; Kaj and Mugal 2016; Torres *et al.* 2020; Müller *et al.* 2022). Applications to selection will likely require free recombination between sites. Desai and Plotkin (2008) applied the independent-Poissons model (for all variant counts in the sample) for example under a version of the Poisson Random Field model (Sawyer and Hartl 1992).

## Figures and Tables

**Fig. A1.**
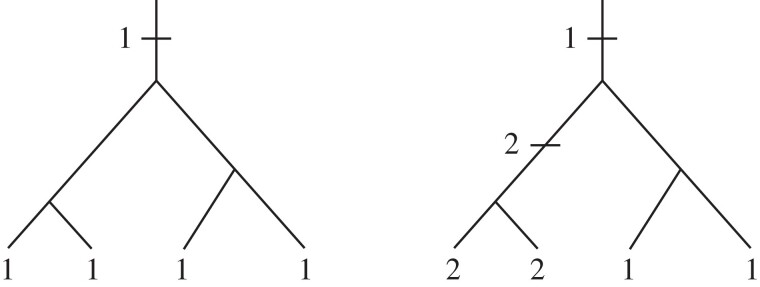
Two subtrees in $\left\{\Gamma_{i}^{\left(4\right)}\right\}$. The subtree on the left has one mutation which is labeled 1 and has count four. The subtree on the right has nested mutations, with the mutation labeled 1 in count two and another labeled 2 also in count two.

## Data Availability

The data application to low-frequency synonymous polymorphisms used allele frequencies from exome sequencing data compiled in gnomAD v2.1.1, available here: https://gnomad.broadinstitute.org/downloads and basepair-resolution mutation rates (Seplyarskiy *et al.* 2022), available here: http://genetics.bwh.harvard.edu/downloads/Vova/Roulette/. The mutation rate model specifies the rate for all three possible alternative nucleotides, and different nucleotide mutations were counted separately when generating the site-frequency spectra. The pipeline used to compile and annotate all potential synonymous mutations in the human genome is available at: https://github.com/vseplyarskiy/Roulette. The site-frequency spectra in different mutation rate bins is available at: https://doi.org/10.6084/m9.figshare.3426251.v1.

## Appendix

### Time-dependent conditional ancestral process

Here we study the conditional ancestral process in detail and provide the justification for (18) and (19).

Let $N_{1}(t)$ and $N_{2}(t)$ be the numbers of rare alleles and common alleles respectively at time *t*. From (17a), (17b) and (17c), the stochastic process $\{(N_{1}(t),N_{2}(t)){\}}_{t\in R_{+}}$ is a continuous-time Markov chain on $Z_{+}^{2}$ with total rate of events $\lambda (n_{1},n_{2})=n_{2}^{2}/2$ and one-step transitions

(A1)
$$(n_{1},n_{2})\to \left\{ \begin{array}{cc} \left(n_{1}-1,n_{2}+1\right) & w/\mathrm{prob}.\;\frac{\theta \pi_{1}}{\theta \pi_{1}+n_{1}-1}\frac{n_{1}}{n_{2}}\\ \left(n_{1}-1,n_{2}\right) & w/\mathrm{prob}.\;\frac{n_{1}-1}{\theta \pi_{1}+n_{1}-1}\frac{n_{1}}{n_{2}}\\ \left(n_{1},n_{2}-1\right) & w/\mathrm{prob}.\;1-\frac{n_{1}}{n_{2}} \end{array}\right.$$

Let $P_{n}$ be the probability measure for this process starting at $n=(n_{1},n_{2})$, and define the random times

(A2)
$$T_{i}\;:=\inf\{t\geq 0\;:\;\;N_{1}(t)=n_{1}-i\}$$

to be the times at which the first coordinate of the process decreases to $n_{1}-i$ for $1\leq i\leq n_{1}$, with $T_{0}=0$. We have $0=T_{0}<T_{1}<T_{2}<\cdots <T_{n_{1}}$ almost surely under $P_{n}$, and the process $\left(N_{1},N_{2}\right)$ visits the following points in order $\left(n_{1},n_{2})\to (n_{1}-1,\;N_{2}(T_{1}))\to \cdots \to (0,\;N_{2}(T_{n_{1}})\right)$.

In Theorem A1 we describe the joint distribution of the hitting times $(T_{i}{)}_{i=1}^{n_{1}}$ and the locations $(N_{2}(T_{i}){)}_{i=1}^{n_{1}}$ as $n_{2}\to \infty$.

Theorem A1 As $n_{2}\to \infty$, the random vector

(A3) $${\left(n_{2}(T_{i}-T_{i-1}),\;\frac{N_{2}(T_{i})}{n_{2}}\right)}_{i=1}^{n_{1}}$$

in $R_{+}^{2n_{1}}$ converges in distribution under $P_{n}$ to the random vector

$${\left(\frac{Z_{i}}{\left(1-Y_{0})(1-Y_{1})\cdots (1-Y_{i-1}\right)},\;(1-Y_{1})\cdots (1-Y_{i})\right)}_{i=1}^{n_{1}},$$

where $Y_{0}=0$, and $\{Y_{i},\;Z_{i}{\}}_{i=1}^{n_{1}}$ are independent random variables with probability density functions

$$\begin{array}{rl} \;f_{Y_{i}}(y)\;= & \left(n_{1}-i+1)(1-y{)}^{n_{1}-i}\;\text{ for}\;y\in (0,1\right)\\ \text{and}\;f_{Z_{i}}(z)\;= & (n_{1}-i+1)\;\frac{2^{n_{1}-i+1}}{(z+2{)}^{n_{1}-i+2}}\;\text{ for}\;z\in (0,\infty ). \end{array}$$

Remark A1 Mean of $T_{n_{1}}$ Note that

$$E[Z_{i}]=\left\{ \begin{array}{cc} \frac{2}{\left(n_{1}-i\right)} & \text{if }1\leq i\leq n_{1}-1\\ \infty & \text{if }i=n_{1} \end{array}\right..$$

Hence for $n_{1}\geq 2$, Theorem A1 implies that $E_{n}[T_{1}]$ is of order $1/n_{2}$ and gives the second part of (18) in the main text. In contrast, when $n_{1}=1$, $E[Z_{1}]=\infty$ and $E_{n}[T_{n_{1}}]$ is no longer of order $1/n_{2}$. Indeed, when $n_{1}=1$,$P_{n}(♯=k)=\frac{1}{n_{2}}$ for $k\in \{0,1,\ldots n_{2}-1\}$ by (A6). Hence by (A4) and Fubini’s theorem,

$$\begin{array}{rl} E_{n}[T_{1}] & =\sum_{k=0}^{n_{2}-1}\sum_{i=0}^{k}E_{n}[\xi_{i}]\;P_{n}(♯=k)\\ & =\sum_{k=0}^{n_{2}-1}\sum_{i=0}^{k}\frac{2}{(n_{2}-i{)}^{2}}\frac{1}{n_{2}}\\ & =\frac{2}{n_{2}}\sum_{i=0}^{n_{2}-1}\frac{1}{\left(n_{2}-i\right)}\\ & \approx \frac{2}{n_{2}}\log\;n_{2}\;\text{as }n_{2}\to \infty . \end{array}$$

These give (18) in the main text.

Remark A2 Mean of $N_{2}(T_{1})$ By (A5) and Theorem A1,

$$\lim_{n_{2}\to \infty }E\left[\frac{N_{2}(T_{i})}{n_{2}}\right]=\frac{n_{1}}{n_{1}+1}\frac{n_{1}-1}{n_{1}}\cdots \frac{n_{1}-i+1}{n_{1}-i+2}=\frac{n_{1}-i+1}{n_{1}+1}$$

for $1\leq i\leq n_{1}$. This gives (19) in the main text.

#### Proof of Theorem A1

To explain the key idea we first establish weak convergence of $\left(n_{2}\;T_{1},\;\frac{N_{2}(T_{1})}{n_{2}}\right)$, i.e. of the marginal distribution for i=1 in (A3). By definition, $T_{1}$ is given by

(A4)
$$T_{1}=\sum_{i=0}^{♯}\xi_{i},$$

where ♯ is the number of downward jumps in second coordinate of the process starting at $\left(n_{1},n_{2}\right)$ up to the first decrease in the first coordinate. The variables $\{\xi_{i}{\}}_{i=0}^{♯-1}$ are the times between these downward jumps, with $\xi_{♯}$ being the time to the final jump starting at $\left(n_{1},n_{2}-♯\right)$. This last jump is the one which decreases the first coordinate. Observe that $N_{2}(T_{1})$ is either $n_{2}-♯$ or $n_{2}-♯+1$. Given ♯, $N_{2}(T_{1})$ is equal to

(A5)
$$\left\{ \begin{array}{cc} n_{2}-♯+1, & w/\mathrm{conditional}\;\mathrm{prob}.\;\frac{\theta \pi_{1}}{\theta \pi_{1}+n_{1}-1}\frac{n_{1}}{n_{2}-♯}\\ n_{2}-♯, & w/\mathrm{conditional}\;\mathrm{prob}.\;\frac{n_{1}-1}{\theta \pi_{1}+n_{1}-1}\frac{n_{1}}{n_{2}-♯} \end{array}\right.$$

which correspond to a non-empty mutation event and a coalescent event of type 1 respectively. These follow from (A1).

The probability mass function of ♯ is given by $P_{n}(♯=0)=\frac{n_{1}}{n_{2}}$ and, for $k\in \{1,2,\ldots ,n_{2}-n_{1}\}$,

(A6)
$$\begin{array}{rl} & P_{n}(♯=k)\\ & \;=\left(1-\frac{n_{1}}{n_{2}}\right)\left(1-\frac{n_{1}}{n_{2}-1}\right)\cdots \left(1-\frac{n_{1}}{n_{2}-k+1}\right)\;\frac{n_{1}}{n_{2}-k}\\ & \;=\frac{n_{1}}{n_{2}}\prod_{\;j=1}^{k}\frac{n_{2}-n_{1}-j+1}{n_{2}-j}\\ & \;=\frac{n_{1}}{n_{2}}\prod_{\;j=1}^{n_{1}-1}\frac{n_{2}-k-j}{n_{2}-j} \end{array}$$

(A7)
$$\;\approx \frac{n_{1}}{n_{2}}\;(1-x{)}^{n_{1}-1}$$

as $n_{2}\to \infty$ and $\frac{k}{n_{2}}\to x\in (0,1)$. Hence $P_{n}(♯>n_{2}-n_{1})=0$ and, for $k\in \{0,1,2,\ldots ,n_{2}-n_{1}-1\}$,

(A8)
$$\begin{array}{rl} P_{n}(♯>k) & =\left(1-\frac{n_{1}}{n_{2}}\right)\left(1-\frac{n_{1}}{n_{2}-1}\right)\cdots \left(1-\frac{n_{1}}{n_{2}-k}\right)\\ & \approx \prod_{\;j=1}^{n_{1}}\left(1-\frac{k+j}{n_{2}}\right)\\ & \approx (1-x{)}^{n_{1}} \end{array}$$

as $n_{2}\to \infty$ and $\frac{k}{n_{2}}\to x\in (0,1)$.

Lemma A1 As $n_{2}\to \infty$, we have convergence in distribution

$$\left(n_{2}\;\sum_{i=0}^{♯}\xi_{i},\;\frac{♯}{n_{2}}\right)\;\overset{d}{⟶}\;(Z_{1},\;Y_{1}).$$

with $Z_{1}$ and $Y_{1}$ as defined in Theorem A1.

Proof of Lemma A1. It suffices to show that the moment generating function of the $R^{2}$-valued random variable on the left converges pointwise to that on the right; that is, to show that

(A9) $$\lim_{n_{2}\to \infty }E_{n}\left[e^{\eta \;\frac{♯}{n_{2}}\;+\;\zeta \;n_{2}T_{1}}\right]=n_{1}\int_{0}^{1}(1-x{)}^{n_{1}-1}e^{\left\{\eta \;x\;+\;\frac{2\zeta x}{1-x}\right\}}\;dx$$

for η∈R and ζ∈(−∞,0]. See, for instance, Section 30 of Billingsley (2008). Since $\xi_{i}∼$ Exp$\left(\lambda (n_{1},n_{2}-i)\right)$,

(A10) $$E_{n}[e^{\zeta \;\xi_{i}}]=\frac{\lambda (n_{1},n_{2}-i)}{\lambda (n_{1},n_{2}-i)-\zeta }=\frac{(n_{2}-i{)}^{2}}{(n_{2}-i{)}^{2}-2\zeta }.$$

By (A4), (A6) and (A10),

(A11) $$\begin{array}{rl} & E_{n}\left[e^{\eta \;\frac{♯}{n_{2}}+\zeta \;n_{2}T_{1}}\right]=\sum_{k=0}^{n_{2}-n_{1}}P_{n}(♯=k)\;e^{\eta \;\frac{k}{n_{2}}}\;E_{n}\left[e^{\zeta n_{2}\sum_{i=0}^{k}\xi_{i}}\right]\\ & =\sum_{k=0}^{n_{2}-n_{1}}P_{n}(♯=k)\;e^{\eta \;\frac{k}{n_{2}}}\prod_{i=0}^{k}E_{n}\left[e^{\zeta \;n_{2}\;\xi_{i}}\right]\\ & =\frac{n_{1}}{n_{2}}\sum_{k=0}^{n_{2}-n_{1}}e^{\eta \frac{k}{n_{2}}}\prod_{\;j=1}^{n_{1}-1}\frac{n_{2}-k-j}{n_{2}-j}\;p_{n_{2}}(\zeta ), \end{array}$$

where

(A12) $$\begin{array}{rl} \;p_{n_{2}}(\zeta ) & \;:=\prod_{i=0}^{k}\frac{\lambda (n_{1},n_{2}-i)}{\lambda (n_{1},n_{2}-i)-\zeta n_{2}}\\ & =\exp\;\left\{-\sum_{i=0}^{k}\log\;\left(1-\frac{2\zeta n_{2}}{(n_{2}-i{)}^{2}}\right)\right\}\\ & \approx \exp\;\left\{2\zeta n_{2}\sum_{i=0}^{k}\frac{1}{(n_{2}-i{)}^{2}}\right\}\;\text{if }\frac{2\zeta n_{2}}{(n_{2}-i{)}^{2}}\approx 0\\ & \approx \exp\;\left\{2\zeta \int_{0}^{x}\frac{1}{(1-y{)}^{2}}\;dy\right\}=\exp\;\left\{\frac{2\zeta x}{1-x}\right\} \end{array}$$

if $\frac{k}{n_{2}}\to x\in (0,1)$ and $n_{2}\to \infty$. Putting (A12) and (A7) into (A11), we obtain the desired (A9) and thus Lemma A1.  □

We now return to the proof of Theorem A1. Lemma A1 implies that $\left(n_{2}\;T_{1},\;N_{2}(T_{1})/n_{2}\right)$ converges in distribution to $\left(Z_{1},1-Y_{1}\right)$ as $n_{2}\to \infty$. Since $Y_{1}<1$ almost surely, we have $N_{2}(T_{1})\to \infty$ in the sense that

(A13)
$$\lim_{n_{2}\to \infty }P_{n}(N_{2}(T_{1})>M)=1\;\text{ for all }M\in (0,\infty ).$$

As in (A4), by definition, $T_{2}$ is given by

$$T_{2}=T_{1}+\sum_{i=0}^{{♯}_{2}}\xi_{i}^{\left(2\right)},$$

where ${♯}_{2}$ is the number of downward jumps starting in state $\left(n_{1}-1,\;N_{2}(T_{1})\right)$ up to the second decrease in the first coordinate, i.e. to $n_{1}-2$. Like before, $\{\xi_{i}^{\left(2\right)}{\}}_{i=0}^{{♯}_{2}-1}$ are the times between these jumps, with $\xi_{{♯}_{2}}^{\left(2\right)}$ being the time for first coordinate to hit $n_{1}-2$ starting at the penultimate states $\left(n_{1}-1,N_{2}(T_{1})-{♯}_{2}\right)$. As in (A5), $N_{2}(T_{2})$ is either $N_{2}(T_{1})-{♯}_{2}$ or $N_{2}(T_{1})-{♯}_{2}+1$.

As $n_{2}\to \infty$, $N_{2}(T_{1})\to \infty$ in the sense of (A13). Hence the same argument that leads to Lemma 1 can be applied again, starting at the new location $\left(n_{1}-1,\;N_{2}(T_{1})\right)$. More precisely, by computing moment generating functions as before, and applying the strong Markov property of the random walk $\{(N_{1}(t),N_{2}(t)){\}}_{t\in R_{+}}$ at the stopping time $T_{1}$, we obtain the joint convergence

$$\left(n_{2}\sum_{i=0}^{♯}\xi_{i},(n_{2}-♯)\sum_{i=0}^{{♯}_{2}}\xi_{i}^{\left(2\right)}\;;\;\frac{♯}{n_{2}},\frac{{♯}_{2}}{n_{2}-♯}\right)\;\overset{d}{⟶}\;(Z_{1},Z_{2}\;;\;Y_{1},Y_{2})$$

under $P_{n}$ as $n_{2}\to \infty$, where $\left\{Z_{1},Z_{2},Y_{1},Y_{2}\right\}$ are independent variables defined in Theorem A1. This implies the convergence in distribution

$$\begin{array}{rl} & \left(n_{2}T_{1},\;n_{2}(T_{2}-T_{1})\;;\;\frac{N_{2}(T_{1})}{n_{2}},\;\frac{N_{2}(T_{2})}{n_{2}}\right)\\ & \;\;\overset{d}{⟶}\;\left(Z_{1},\frac{Z_{2}}{1-Y_{1}}\;;\;1-Y_{1},\;(1-Y_{1})(1-Y_{2})\right) \end{array}$$

under $P_{n}$, as $n_{2}\to \infty$. Continuing this way, by letting ${♯}_{i}$ be the number of downward jumps starting at $\left(n_{1}-i+1,\;N_{2}(T_{i-1})\right)$ before hitting the vertical line $\left\{(n_{1}-i,y)\;:\;\;y\in Z_{+}\right\}$ for i≥1, we obtain the desired convergence in Theorem A1.  □

### Low-count branches of general coalescent trees

Here we prove the non-nestedness and Poisson-independence of low-count mutations, which we assumed in section “Theory for nonconstant populations.” We do this first for fixed trees then for the random, general coalescent trees of Griffiths and Tavaré (1998). We also present the computation of the probability generating function, $G_{n,k}(x,y)$, of the count of the variant of interest and its number of latent mutations. Our definition of nested differs from some previous ones (Saunders *et al.* 1984; Wiuf and Donnelly 1999; Hobolth and Wiuf 2009); here nested mutations may occur on the same branch of the gene genealogy.

#### Nested mutation on a fixed tree

Let $T_{n}$ be a fixed (non random) tree with *n* leaves. We suppose the tree is ultrametric, that is the leaves have the same distance $H_{n}$ from the root. We call $H_{n}$ the height of $T_{n}$. Consistent with the main text, we adopt the following notation for some relevant properties of $T_{n}$, for the most part suppressing the dependence on *n* for simplicity:

1. $T_{k}$ is the length of the time during which there are exactly *k* lineages ancestral to the sample, for k∈{2,3,…,n}.
2. $\tau_{j}$ for j∈{1,…,n−1}, is the total length of branches in $T_{n}$ that have *j* descendants. We suppose there are $m_{j}$ such branches with lengths $\{\tau_{\;j,k}{\}}_{k=1}^{m_{j}}$. Then $\tau_{j}=\sum_{k=1}^{m_{j}}\tau_{\;j,k}$.
3. $T_{\mathrm{total}}$ is the total branch length, the sum of all the branches in $T_{n}$, which is equal to $\sum_{k=2}^{n}k\;T_{k}=\sum_{\;j=1}^{n-1}\tau_{j}$.
4. For a positive integer *b*, we define a collection $\{\Gamma_{i}^{\left(b\right)}{\}}_{i=1}^{m_{b}}$ of disjoint connected subtrees of the coalescent tree as follows: Each of the $m_{b}$ branches with *b* descendants in the sample (say the *i*th one) subtends *b* leaves in the coalescent tree and gives rise to a subtree $\Gamma_{i}^{\left(b\right)}$ which contains that branch. We say **nested mutation up to count *b*** occurs on $T_{n}$ if there exist two mutations on $\Gamma_{i}^{\left(b\right)}\subset T_{n}$ for some $i\in \{1,2,\ldots ,m_{b}\}$. Fig. A1 illustrates this for b=4.

We assume that mutations arise as a Poisson point process on the tree with constant rate θ/2 per unit length. Theorem A2 below holds for any fixed ultrametric tree (it can be binary or have multiple mergers, or even be a star tree).

Theorem A2 Nested mutation on fixed trees Let $T_{n}$ be a fixed ultrametric tree with *n* leaves. For any positive integer *b* and for any θ∈(0,∞), the probability that nested mutation up to count *b* occurs is bounded above by

(A14) $$\min\left\{\frac{\theta^{2}}{8}b\;T_{\mathrm{total}}\;H_{n},\;\frac{\theta^{2}}{8}\;b^{3}\;\sum_{\;j=1}^{b}\sum_{k=1}^{m_{j}}\tau_{\;j,k}^{2}\right\}.$$

In particular, the probability that nested mutation up to count *b* occurs tends to 0, as n→∞, if $\theta^{2}(\max_{1\leq k\leq m_{j}}\tau_{\;j,k})\tau_{j}\to 0$ for 1≤j≤b.

Remark A3 There is good evidence that the upper bound $\frac{\theta^{2}}{8}T_{\mathrm{total}}\;H_{n}$ is actually small for humans. For the gnomAD data we analyze in the main text, the expected number of mutations per site ($\theta T_{\mathrm{total}}/2$) is between about 0.009 and 2.13. So $\theta T_{\mathrm{total}}/2$ is not big with high probability. The rest of the upper bound, $b\theta H_{n}/4$, should be proportional to the average pairwise difference per site (very nearly equal to this for random Kingman coalescent trees and large *n*) and this ranges from about $9\times 10^{-5}$ to about 0.02 for these same data. See section “Application to human SNP data.”

Remark A4 The simpler bound $\frac{\theta^{2}}{8}bT_{\mathrm{total}}H_{n}$ can be weaker than the other bound $\frac{\theta^{2}}{8}b^{3}\sum_{\;j=1}^{b}\sum_{k=1}^{m_{j}}\tau_{\;j,k}^{2}$ in (A14) for large *n*. For the Kingman coalescent, $E[T_{\mathrm{total}}H_{n}]=O(\log\;n)$ is larger than $E[\sum_{\;j=1}^{b}\sum_{k=1}^{m_{j}}\tau_{\;j,k}^{2}]$ since the latter tends to 0 as n→∞, by (A17). For a star tree, however, both bounds are approximately $\theta^{2}nH_{n}^{2}$ (up to a multiplicative constant).

Proof. The total number $M_{n}$ of mutations on $T_{n}$ is a Poisson variable with mean $c_{n}\;:=\frac{\theta }{2}T_{\mathrm{total}}$. Given the tree $T_{n}$ and $M_{n}=k$, the *k* mutations are uniformly distributed on the tree. Hence the conditional probability that two given mutations are on the same subtree $\Gamma_{i}^{\left(b\right)}$ for some *i* is equal to

$$\sum_{i=1}^{m_{b}}\frac{{\left|\Gamma_{i}^{\left(b\right)}\right|}^{2}}{T_{\mathrm{total}}^{2}},$$

where $\left|\Gamma_{i}^{\left(b\right)}\right|$ is the total branch lengths of the subtree $\Gamma_{i}^{\left(b\right)}$. Since there are k(k−1)/2 ways to choose two mutations out of *k*,

(A15) $$\begin{array}{rl} & \;P(\text{there are 2 mutations on }\Gamma_{i}^{\left(b\right)}\text{ for some }i\in \{1,2,\ldots ,m_{b}\})\\ & \;\leq \sum_{k=0}^{\infty }e^{-c_{n}}\frac{c_{n}^{k}}{k!}\frac{k(k-1)}{2}\sum_{i=1}^{m_{b}}\frac{|\Gamma_{i}^{\left(b\right)}{|}^{2}}{T_{\mathrm{total}}^{2}}\\ & \;=\frac{c_{n}^{2}}{2}\sum_{i=1}^{m_{b}}\frac{|\Gamma_{i}^{\left(b\right)}{|}^{2}}{T_{\mathrm{total}}^{2}}\\ & \;=\frac{\theta^{2}}{8}\sum_{i=1}^{m_{b}}|\Gamma_{i}^{\left(b\right)}{|}^{2}. \end{array}$$

Note that $|\Gamma_{i}^{\left(b\right)}|\leq bH_{n}$ for all $1\leq i\leq m_{b}$, and that $\sum_{i=1}^{m_{b}}|\Gamma_{i}^{\left(b\right)}|\leq T_{\mathrm{total}}$ since the subtrees $\{\Gamma_{i}^{\left(b\right)}{\}}_{i=1}^{m_{b}}$ are disjoint. Hence

$$\sum_{i=1}^{m_{b}}|\Gamma_{i}^{\left(b\right)}{|}^{2}\leq bH_{n}\sum_{i=1}^{m_{b}}|\Gamma_{i}^{\left(b\right)}|\leq bT_{\mathrm{total}}H_{n}.$$

Putting this into (A15), we obtained the first bound $\frac{\theta^{2}}{8}bT_{\mathrm{total}}H_{n}$ in (A14). To get the second bound in (A14), note that $|\Gamma_{i}^{\left(b\right)}|\leq bH_{i}^{\left(b\right)}$ for all $1\leq i\leq m_{b}$, where $H_{i}^{\left(b\right)}$ is the height of the subtree $\Gamma_{i}^{\left(b\right)}$. Furthermore, $H_{i}^{\left(b\right)}$ is the sum of at most *b* branch lengths, one from $\left\{\tau_{\;j,k}\right\}$ for j=b,b−1,…,2,1, and these branches are pairwise disjoint for different *i*’s (for $1\leq i\leq m_{b}$). Hence

$$\sum_{i=1}^{m_{b}}|H_{i}^{\left(b\right)}{|}^{2}\leq b\sum_{\;j=1}^{b}\sum_{k=1}^{m_{j}}\tau_{\;j,k}^{2},$$

where we used the general inequality $|\sum_{k=1}^{b}a_{k}{|}^{2}\leq b\sum_{k=1}^{b}a_{k}^{2}$. The bound in (A14) now follows by putting these into (A15).  □

A mutation on a tree (called a latent mutation in the main text) is said to **have count *j*** if the mutation is the most recent mutation in the lineages of exactly *j* individuals at the leaves of the tree; see Fig. A1.

Theorem A3 Poisson approximation for counts on a fixed tree Let $T_{n}$ be a fixed coalescent tree with *n* leaves for n≥2. Let $a_{j}$ be the number of mutations on $T_{n}$ with counts *j*. If the probability that nested mutation up to count *b* occurs tends to 0 as n→∞, then for any positive integer *b* and any θ∈(0,∞), the variables $\{a_{j}{\}}_{\;j=1}^{b}$ are asymptotically independent and $a_{j}∼\mathrm{Poisson}(\frac{\theta }{2}\tau_{j})$ for 1≤j≤b.

Proof. If there is no nested mutation up to count *b*, then $a_{j}$ is also equal to the number of mutations on the branches in $T_{n}$ that have *j* descendants, for 1≤j≤b. Since these branches have total length $\tau_{j}$ and they are disjoint for different *j*’s, the result follows from the assumption that mutations occur as a Poisson point process on the tree $T_{n}$ with rate θ/2.  □

#### Nested mutation on random trees

We now suppose the tree $T_{n}$ is a *random* binary tree (for n≥2), in particular the general coalescent tree of Griffiths and Tavaré (1998). For each n≥2, $\{T_{k}{\}}_{k=2}^{n}$ is a sequence of positive random variables representing the times during which there are *k* lineages in $T_{n}$. The branching structure of $T_{n}$ is independent of the times $\{T_{k}{\}}_{k=2}^{n}$. Looking forward in time, whenever there is a branching event, an existing lineage is chosen uniformly at random to split into two.

Following Griffiths and Tavaré (1998, eqn. (2.2)) we let λ(t) be the the population size at time *t* in the past divided by the current population size. As in (45), $\lambda (t)=e^{-\beta t}$ with β>0 corresponds to an exponentially growing population.

Theorem A4 Nested mutation on random trees for fixed θ Let b∈N. Suppose for 1≤j≤b,

(A16) $$\lim_{n\to \infty }E_{n}\left[\sum_{k=1}^{m_{j}}\tau_{\;j,k}^{2}\right]=0,$$

where the expectation $E_{n}$ averages over all realizations of $T_{n}$. Then the probability that nested mutation up to count *b* occurs is bounded above by $C_{b,n}\theta^{2}$, where $\{C_{b,n}{\}}_{n\geq 2}$ are constants that tend to 0 as n→∞. Furthermore, (A16) holds for the generalized coalescent trees of Griffiths and Tavaré (1998) when $\underset{t\geq 0}{\sup}\lambda (t)<\infty$ (which includes any growing population).

Proof. The first statement follows directly from Theorem A2. By the fact $\sum_{k=1}^{m_{j}}\tau_{\;j,k}^{2}\leq (\max_{1\leq k\leq m_{j}}\tau_{\;j,k})\tau_{j}$ and the Cauchy-Schwarz inequality, we have

(A17) $$E_{n}\left[\sum_{k=1}^{m_{j}}\tau_{\;j,k}^{2}\right]\leq \sqrt{E_{n}[\tau_{j}^{2}]E_{n}\left[{\left(\max_{1\leq k\leq m_{j}}\tau_{\;j,k}\right)}^{2}\right]}.$$

Hence assumption (A16) is satisfied if

(A18) $$\lim_{n\to \infty }E_{n}\left[{\left(\max_{1\leq k\leq m_{j}}\tau_{\;j,k}\right)}^{2}\right]=0$$

(A19) $$\underset{n\to \infty }{\text{lim sup}}\;E_{n}\left[\tau_{j}^{2}\right]<\infty ,$$

for 1≤j≤b. The second statement now follows from Lemmas A2, A3, and Proposition A1 below.  □

Lemma A2 concerns assumption (A18). For reference, we note that it is satisfied, and hence (A18) is satisfied, if $T_{k}$ are exponential variables with parameter $\lambda_{k}$ where $\sum_{k=2}^{\infty }\frac{1}{\lambda_{k}}<\infty$. This is true for the Kingman coalescent which has $\lambda_{k}=k(k-1)/2$.

Lemma A2 Suppose ${\text{lim sup}}_{n\to \infty }\sum_{k=2}^{n}T_{k}$ has finite *p*th moment, where p>0. Then $\max_{1\leq k\leq m_{j}}\tau_{\;j,k}\to 0$ in $L^{p}$, as n→∞.

Proof. Consider the random tree $T_{n}$ and recall that $T_{k}$ is the length of the time during which there are exactly *k* lineages ancestral to the sample in $T_{n}$. These *k* lineages are segments of length $T_{k}$ of the branches of the genealogy, and each of them is called a line of state *k*. We construct the infinite sequence $\{T_{n}{\}}_{n\geq 2}$ sequentially in the same probability space, by constructing a coupling of the two independent families $\{T_{k}{\}}_{k\geq 2}$ and $\{\iota_{k}{\}}_{k\geq 2}$, where $\iota_{n}\in \{1,2,\ldots ,n\}$ is the index of the lineage that branches into two going from $T_{n}$ to $T_{n+1}$. Let $A_{\ell }^{\left(k,n\right)}$ be the number of descendants in $T_{n}$ of the ℓth line of state *k*. Note that $A_{\ell }^{\left(k,n\right)}\geq 1$ for ℓ∈{1,2,…,k}, and $\sum_{\ell =1}^{k}A_{\ell }^{\left(k,n\right)}=n$. By exchangeability—in particular see Bertoin (2006, Proposition 2.8)—the random vector $\frac{1}{n}(A_{1}^{\left(k,n\right)},\ldots ,A_{k}^{\left(k,n\right)})$ converges almost surely to a random vector that has the symmetric Dirichlet distribution on the simplex $\left\{(x_{i}{)}_{i=1}^{k}\in R_{+}^{k}\;:\;\;x_{1}+\cdots +x_{k}=n\right\}$. Therefore, with probability one,

(A20) $$\lim_{n\to \infty }A_{\ell }^{\left(k,n\right)}=+\infty \;\text{ for all }k\geq 1\text{ and }\ell =1,2,\ldots ,k.$$

Since $\sum_{k=2}^{\infty }T_{k}$ is finite almost surely, the trees $\{T_{n}{\}}_{n\geq 2}$ have uniformly bounded height almost surely. So (A20) implies that with probability one,

$${\text{lim sup}}_{\;n\to \infty }\;\underset{1\leq k\leq m_{j}}{\sup}\tau_{\;jk}=0\;\text{ for all }j\geq 1.$$

Since $\max_{1\leq k\leq m_{j}}\tau_{\;j,k}<\sum_{k=2}^{n}T_{k}$, by the assumption on $\left\{T_{k}\right\}$ and the Dominated Convergence Theorem, $\max_{1\leq k\leq m_{j}}\tau_{\;j,k}\to 0$ in $L^{p}$ as n→∞.  □

Next consider assumption (A19). For the Kingman coalescent, $\tau_{j}$ is close to its mean $E_{n}[\tau_{j}]=2/j$ because for *n* large enough,

(A21)
$$\mathrm{Var}(\tau_{j})=4\sigma_{\;jj}\leq \frac{4(j+1)\log\;n}{n},$$

where $\sigma_{\;jj}$ is defined in Fu (1995, eqns. (1)-(2)). This follows from the fact that Fu’s $\beta_{n}(j)\approx \frac{2\log\;n}{n}$ as n→∞ for each j≥1 (Fu 1995, eqn. (5)). Hence

$${\text{lim sup}}_{\;n\to \infty }\;E_{n}[\tau_{j}^{2}]\leq {\left(\frac{2}{j}\right)}^{2}.$$

Lemma A3 Suppose there exists a constant $C_{*}\in (0,\infty )$ such that

(A22) $$\underset{n\geq 2}{\sup}E_{n}[T_{k}^{2}]\leq \frac{C_{*}}{k^{4}}\;\text{ for all }k\geq 2.$$

Then $E_{n}[\tau_{j}]\leq \frac{\sqrt{C_{*}}}{j}$ for all j≥1 and ${\text{limsup}}_{n\to \infty }E_{n}[\tau_{j}^{2}]<\infty$.

Proof. For realized values of $T_{k}$, the argument in Fu (1995, p. 181) gives

$$\tau_{j}=\sum_{k=2}^{n}\sum_{\ell =1}^{k}\epsilon_{k,\ell }(j)\;T_{k}=\sum_{k=2}^{n}T_{k}\sum_{\ell =1}^{k}\epsilon_{k\ell }(j),$$

where $\epsilon_{k\ell }(j)=1_{\left\{A_{\ell }^{\left(k,n\right)}=j\right\}}$ is the indicator variable, where $A_{\ell }^{\left(k,n\right)}$ is the number of descendants in $T_{n}$ of the ℓth line of state *k* defined in the proof of Lemma A2. Using the independence between $\{T_{k}{\}}_{k\geq 2}$ and the branching structure, and following the notation in Fu (1995, eqns. (18)-(19)), the conditional expectation of $\tau_{j}$, given $\{T_{k}{\}}_{k=2}^{n}$, is

(A23) $$E_{n}[\tau_{j}\;|\;\{T_{k}{\}}_{k=2}^{n}]=\sum_{k=2}^{n}T_{k}\;k\;p(k,j)$$

and that of $\tau_{j}^{2}$, given $\{T_{k}{\}}_{k=2}^{n}$, is

(A24) $$\begin{array}{rl} E_{n}[\tau_{j}^{2}\;|\;\{T_{k}{\}}_{k=2}^{n}] & =\sum_{k=2}^{n}T_{k}^{2}(kp(k,j)+k(k-1)p(k,j;k,j))\\ & +2\sum_{k<k^{\prime }}T_{k}T_{k^{\prime }}kk^{\prime }p(k,j;k^{\prime },j), \end{array}$$

where the deterministic functions $p(k,j),\;p(k,j;k^{\prime },j)$ do not depend on $\left\{T_{k}\right\}$. From Fu (1995),

$$p(k,j)=\frac{\left(\begin{array}{c} n-j-1\\ k-2 \end{array}\right)}{\left(\begin{array}{c} n-1\\ k-1 \end{array}\right)}=\frac{\left(\begin{array}{c} n-k\\ \;j-1 \end{array}\right)}{\left(\begin{array}{c} n-1\\ \;j \end{array}\right)}\frac{k-1}{j},\;p(k,j;k,j)=\frac{\left(\begin{array}{c} n-2j-1\\ k-3 \end{array}\right)}{\left(\begin{array}{c} n-1\\ k-1 \end{array}\right)}$$

and for $2\leq k<k^{\prime }\leq n$,

$$\begin{array}{rl} \;p(k,j;k^{\prime },j) & =\frac{k-1}{k^{\prime }(k^{\prime }-1)}p(k^{\prime },j)\\ & \;+\sum_{t}\frac{\left(\begin{array}{c} k^{\prime }-k\\ t-1 \end{array}\right)}{\left(\begin{array}{c} k^{\prime }-1\\ t \end{array}\right)}\frac{\left(k-1)(k^{\prime }-t\right)}{tk^{\prime }}\frac{\left(\begin{array}{c} \;j-1\\ t-1 \end{array}\right)\left(\begin{array}{c} n-2j-1\\ k^{\prime }-2-t \end{array}\right)}{\left(\begin{array}{c} n-1\\ k^{\prime }-1 \end{array}\right)}, \end{array}$$

where the sum is taken over $1\leq t\leq \min\{j,\;k^{\prime }-2,\;k^{\prime }-k+1\}$. The first and the second moments of $\tau_{i}$ are obtained averaging over $\{T_{k}{\}}_{k=2}^{n}$ in (A23) and (A24). The bound $E_{n}[\tau_{j}]\leq \frac{\sqrt{C_{*}}}{j}$ follows from the same calculation in Fu (1995, eqn. (22)). By (A24), the fact $E_{n}[T_{k}T_{k^{\prime }}]\leq (E_{n}[T_{k}^{2}]\;E_{n}[T_{k^{\prime }}^{2}]{)}^{1/2}$ and assumption (A22), ${\text{lim sup}}_{n\to \infty }E_{n}[\tau_{j}^{2}]<\infty$ holds also for our random trees.  □

Remark A5 As in Theorem A2, we can use an alternate assumption than A16. For any positive integer *b*, the probability that nested mutation up to count *b* occurs is bounded above by $\frac{b\theta^{2}}{8}E_{n}[T_{\mathrm{total}}\;H_{n}]$ which tends to 0 if $\theta^{2}E_{n}[T_{\mathrm{total}}H_{n}]\to 0$. For Kingman coalescent trees, this would require that θ→0.

We now check that the assumption (A16) in Theorem A4 holds for the generalized coalescent tree of Griffiths and Tavaré (1998).

Proposition A1 Suppose $C_{0}\;:=\underset{t\geq 0}{\sup}\lambda (t)<\infty$. Then $\left\{T_{k}\;:\;\;2\leq k\leq n,\;n\geq 2\right\}$ satisfy the conditions in both Lemma A2 (with p=2) and Lemma A3. In particular, (A16) is satisfied and so the conclusion of Theorem A4 holds.

Proof. The joint distribution of $\{T_{k}{\}}_{k=2}^{n}$ is determined by the function λ; see Griffiths and Tavaré (1994b). We can construct $\{T_{k}{\}}_{k=2}^{n}$ in terms of λ as follows: let $\{D_{n}(t){\}}_{t\in R_{+}}$ be a pure death process with rate (k2) at state k∈{1,2,…,n}, starting at $D_{n}(0)=n$, and let

(A25) $$D_{n}^{\left(\lambda \right)}(t)=D_{n}\left(\int_{0}^{t}\frac{1}{\lambda (u)}\;du\right)$$

be a time-changed pure death process. Then

$$T_{k}=\int_{0}^{\infty }1\{D_{n}^{\left(\lambda \right)}(t)=k\}\;dt=\sigma_{n-k+1}-\sigma_{n-k},$$

for 2≤k≤n, where $\sigma_{1}<\sigma_{2}<\cdots <\sigma_{n-1}$ are the jump times of $D_{n}^{\left(\lambda \right)}$ (by convention $\sigma_{0}=0$). By (A25), the jump times of the pure death process $D_{n}$, denoted by ${\tilde{\sigma }}_{1}<{\tilde{\sigma }}_{2}<\cdots <{\tilde{\sigma }}_{n-1}$, are given by $\int_{0}^{\sigma_{j}}\frac{1}{\lambda }={\tilde{\sigma }}_{j}$ for 1≤j≤n−1. Hence, with the convention $\sigma_{0}=0$, for 0≤j≤n−2 we have

$$\frac{\sigma_{\;j+1}-\sigma_{\;j}}{C_{0}}\leq \int_{\sigma_{j}}^{\sigma_{\;j+1}}\frac{1}{\lambda (t)}\;dt={\tilde{\sigma }}_{\;j+1}-{\tilde{\sigma }}_{\;j}.$$

These give $T_{k}=\sigma_{n-k+1}-\sigma_{n-k}\leq ({\tilde{\sigma }}_{n-k+1}-{\tilde{\sigma }}_{n-k})C_{0}$ for all 2≤k≤n. Since ${\tilde{\sigma }}_{n-k+1}-{\tilde{\sigma }}_{n-k}$ is equal in distribution to the analog of $T_{k}$ for the Kingman coalescent, $T_{k}$ is stochastically dominated by $C_{0}$ times an exponential variable with parameter k(k−1)/2 for all 2≤k≤n. The desired statement now follows since (A18) and (A19) are satisfied.  □

#### Replacing $\tau_{j}$ by its mean

By using the expected coalescence times denoted ${\bar{\tau }}_{i}$ in the main text, we implicitly assumed that different sites have different trees and that these are all drawn from the same distribution. Theorem A5 below asserts that even though the mutant counts at each site are conditional on the realization of the tree at that site, we can replace $\tau_{j}$ by its expectation $E_{n}[\tau_{j}]$ in Theorem A3 when the trees are random and satisfy suitable assumptions. The key reason is that $\tau_{j}$ is close to its mean, as made precise in Lemma A4.

Lemma A4 Suppose (A22) holds and that the covariance

(A26) $$\mathrm{Cov}(T_{k},T_{k^{\prime }})\leq \frac{C_{n}}{k(k-1)k^{\prime }(k^{\prime }-1)}$$

for $2\leq k<k^{\prime }\leq n$ and n≥2, where $\left\{C_{n}\right\}$ is a sequence that tends to 0 as n→∞. Then for each j≥1, the variance $\mathrm{Var}(\tau_{j})\to 0$ as n→∞. In particular, $|\tau_{j}-E[\tau_{j}]\;|\to 0$ in $L^{2}(P)$ as n→∞.

Proof. By further taking expectations in (A23) and (A24) with respect to $E_{n}$, we obtain the variance

(A27) $$\begin{array}{rl} \mathrm{Var}(\tau_{j}) & =E_{n}[\tau_{j}^{2}]-(E_{n}[\tau_{j}]{)}^{2}\\ & =2\sum_{k<k^{\prime }}kk^{\prime }(E_{n}[T_{k}T_{k^{\prime }}]p(k,j;k^{\prime },j)\\ & \;-E_{n}[T_{k}]E_{n}[T_{k^{\prime }}]p(k,j)p(k^{\prime },j)) \end{array}$$

up to an $O(\frac{\log\;n}{n})$ term. This follows from Fu (1995, eqns. (24)-(25)) and assumption (A22) in Lemma A3. This also leads to (A21). By assumptions (A22) and (A26), the double sum in (A27) is bounded above by

(A28) $$C_{n}\sum_{k<k^{\prime }}\frac{\;p(k,j;k^{\prime },j)}{\left(k-1)(k^{\prime }-1\right)}+C_{*}\sum_{k<k^{\prime }}\frac{\;p(k,j;k^{\prime },j)-p(k,j)p(k^{\prime },j)}{\left(k-1)(k^{\prime }-1\right)}.$$

By Fu (1995, eqns. (29) and (22)), the first and second terms of (A28) are of order o(n) and $O(\frac{\log\;n}{n})$, respectively, as n→∞ for each j≥1. The completes the proof of $\lim_{n\to \infty }\mathrm{Var}(\tau_{j})=0$. The latter implies, by Chebyshev’s inequality, that $\tau_{j}-E[\tau_{j}]\to 0$ in $L^{2}$ as n→∞.  □

Theorem A5 Poisson approximation for counts across loci Let $\{T_{n}{\}}_{n\geq 2}$ be a sequence of random coalescent trees which are the generalized coalescent trees of Griffiths and Tavaré (1998). Suppose $\underset{t\geq 0}{\sup}\lambda (t)<\infty$ and assumption (A26) holds. Let $a_{j}$ be the number of mutations on $T_{n}$ with counts *j*. Then for any positive integer *b* and any θ∈(0,∞), the variables $\{a_{j}{\}}_{\;j=1}^{b}$ are asymptotically independent and $a_{j}∼\mathrm{Poisson}(\frac{\theta }{2}\;E_{n}[\tau_{j}])$ for 1≤j≤b, as n→∞.

Proof. By Theorem A4, the probability that nested mutation up to count *b* occurs tends to 0 as n→∞. The result then follows from Lemma A4 and Theorem A3.  □

It can be checked that exponentially growing popolations clearly satisfy $\underset{t\geq 0}{\sup}\lambda (t)<\infty$ and also assumption (A26). The conclusions of Theorems A4 and A5 then hold for the generalized coalescent trees of Griffiths and Tavaré (1998) when $\lambda (t)=e^{\beta t}$ for $t\in R_{+}$ for some β>0.

Equipped with Theorem A5, we write ${\bar{\tau }}_{i}=E_{n}[\tau_{i}]$ as in the main text and compute the probability generating function $G_{n,k}$ of the count of the variant of interest and its number of latent mutations. The count of the variant of interest is $n=\sum_{i}ia_{i}$ and its number of latent mutations is $k=\sum_{i}a_{i}$. Hence

(A29)
$$\begin{array}{rl} G_{n,k}(x,y) & =\sum_{\left(a_{1},a_{2},\ldots \right)}P(a_{1},a_{2},\ldots )x^{n}y^{k}\\ & =e^{-\frac{\theta }{2}\sum_{i}{\bar{\tau }}_{i}}\sum_{\left(a_{1},a_{2},\ldots \right)}x^{\sum ia_{i}}y^{\sum a_{i}}\prod_{i\geq 1}\frac{(\theta {\bar{\tau }}_{i}/2{)}^{a_{i}}}{a_{i}!}\\ & =e^{-\frac{\theta }{2}\sum_{i}{\bar{\tau }}_{i}}\sum_{\left(a_{1},a_{2},\ldots \right)}\prod_{i\geq 1}\frac{x^{ia_{i}}y^{a_{i}}(\theta {\bar{\tau }}_{i}/2{)}^{a_{i}}}{a_{i}!}\\ & =e^{-\frac{\theta }{2}\sum_{i}{\bar{\tau }}_{i}}\prod_{i\geq 1}\sum_{a_{i}\geq 0}\frac{x^{ia_{i}}y^{a_{i}}(\theta {\bar{\tau }}_{i}/2{)}^{a_{i}}}{a_{i}!}\\ & =e^{-\frac{\theta }{2}\sum_{i}{\bar{\tau }}_{i}}\prod_{i\geq 1}e^{x^{i}y\theta {\bar{\tau }}_{i}/2}\\ & =e^{-\frac{\theta }{2}\sum_{i}{\bar{\tau }}_{i}}e^{\frac{\theta }{2}y\sum_{i}x^{i}{\bar{\tau }}_{i}}\\ & =e^{-\frac{\theta }{2}\sum_{i}{\bar{\tau }}_{i}}\sum_{k=0}^{\infty }\frac{(\frac{\theta }{2}{)}^{k}y^{k}}{k!}{\left(\sum_{i}x^{i}{\bar{\tau }}_{i}\right)}^{k} \end{array}$$

as declared in the main text.

#### A remark on the total number of mutations for large $n_{1}$

The stable probabilities observed in the lower three panels of Fig. 6 and in Fig. 4b suggest that the conditional distribution of $k_{1}$ given $n_{1}$ and a very large *n* will approach a distribution as $n_{1}$ gets larger. That this is the case under constant population size follows from the fact, here as in “A conditional ancestral process for rare variants,” that the number of alleles in the Ewens sampling formula is the sum of independent Bernoulli trials (Arratia *et al.* 1992, 2000). The limiting large-$n_{1}$ distribution is Poisson, but shifted because there must be at least one mutation to produce $n_{1}>0$ copies, so it is $k_{1}-1$ that is Poisson. See Proposition 3.1 of Yamato (2017).

By the following heuristic argument, we suggest that this result holds more broadly, in particular for growing populations or ones in which ${\bar{\tau }}_{i}$ decreases at least as fast with *i* as in the constant-size model, whatever the reason. In this case, when a mutation occurs it will very likely produce a low-count variant because ${\bar{\tau }}_{i}/\sum_{i}{\bar{\tau }}_{i}$ for small *i* will be much greater than ${\bar{\tau }}_{n_{1}}/\sum_{i}{\bar{\tau }}_{i}$ for large $n_{1}$. A large-$n_{1}$ variant which is due for example to $k_{1}=2$ latent mutations will very likely have a count pattern such as $\left(a_{1}=1,a_{n_{1}-1}=1\right)$ or $\left(a_{2}=1,a_{n_{1}-2}=1\right)$ and very unlikely to have one such as $\left(a_{n_{1}/2-j}=1,a_{n_{1}/2+j}=1\right)$ for some small *j*.

Then we expect the probability (31) of seeing $n_{1}$ copies given $k_{1}$ latent mutations to be close to

$$p(n_{1}|k_{1};n\;\mathrm{large},\tau )\approx k_{1}\frac{{\bar{\tau }}_{n_{1}}}{\sum_{i=1}^{n-1}{\bar{\tau }}_{i}}\;\mathrm{when}\;n_{1}\;\mathrm{is}\;\mathrm{large},$$

because each of the $k_{1}$ mutations has a small chance ${\bar{\tau }}_{n_{1}}/\sum_{i}{\bar{\tau }}_{i}$ of producing an appropriately large number of copies, the other $k_{1}-1$ mutations being inconsequential to the total count. Multiplying by the Poisson distribution of $k_{1}$ in (30) and rearranging gives

$$\begin{array}{rl} \;p(n_{1},k_{1};n\;\mathrm{large},\tau ) & \approx k_{1}\frac{{\bar{\tau }}_{n_{1}}}{\sum_{i=1}^{n-1}{\bar{\tau }}_{i}}\frac{(\frac{\theta \pi_{1}}{2}\sum_{i=1}^{n-1}{\bar{\tau }}_{i}{)}^{k_{1}}}{k_{1}!}e^{-\frac{\theta \pi_{1}}{2}\sum_{i=1}^{n-1}{\bar{\tau }}_{i}}\\ & =\frac{\theta \pi_{1}}{2}{\bar{\tau }}_{n_{1}}\cdot \frac{(\frac{\theta \pi_{1}}{2}\sum_{i=1}^{n-1}{\bar{\tau }}_{i}{)}^{k_{1}-1}}{(k_{1}-1)!}e^{-\frac{\theta \pi_{1}}{2}\sum_{i=1}^{n-1}{\bar{\tau }}_{i}} \end{array}$$

for large $n_{1}$. To the left of the · is a probability like (7). To the right of the · is the shifted Poisson distribution, which implicitly averages over the (small) sizes of the $k_{1}-1$ additional mutations.
