# Editor's Note on ‘Recurrent mutation in the ancestry of a rare variant’

**DOI:** 10.1093/genetics/iyad082

**Published:** 2023-05-18

This is an Editor's Note on: *Genetics*, 2023, iyad049, https://doi.org/10.1093/genetics/iyad049

This Version of Record incorporates edits made by the authors prior to manuscript acceptance in response to reviewer feedback, but that were not included in the version that posted as an Accepted Manuscript. The authors, editors, and publisher apologize for any confusion caused to readers.

